# Identification of Gut Microbiome Signatures Associated with Indole Pathway in Tryptophan Metabolism in Patients Undergoing Hemodialysis

**DOI:** 10.3390/biom14060623

**Published:** 2024-05-24

**Authors:** Jih-Kai Huang, Ping-Hsun Wu, Zhao-Feng Chen, Po-Yu Liu, Cheng-Chin Kuo, Yun-Shiuan Chuang, Meng-Zhan Lu, Mei-Chuan Kuo, Yi-Wen Chiu, Yi-Ting Lin

**Affiliations:** 1 Department of Emergency Medicine, Kaohsiung Medical University Hospital, Kaohsiung Medical University, Kaohsiung 807, Taiwan; eric86425@gmail.com; 2 Faculty of Medicine, College of Medicine, Kaohsiung Medical University, Kaohsiung 807, Taiwan; 970392@kmuh.org.tw (P.-H.W.); mechku@kmu.edu.tw (M.-C.K.); chiuyiwen@kmu.edu.tw (Y.-W.C.); 3 Division of Nephrology, Department of Internal Medicine, Kaohsiung Medical University Hospital, Kaohsiung Medical University, Kaohsiung 807, Taiwan; 4 Center for Big Data Research, Kaohsiung Medical University, Kaohsiung 807, Taiwan; kinkipag@gmail.com; 5 Research Center for Precision Environmental Medicine, Kaohsiung Medical University, Kaohsiung 807, Taiwan; 6 Department of Horticulture and Landscape Architecture, National Taiwan University, Taipei 10617, Taiwan; ivan.chen1966@gmail.com; 7 School of Medicine, College of Medicine, National Sun Yat-sen University, Kaohsiung 804, Taiwan; 8 Institute of Cellular and System Medicine, National Health Research Institutes, Zhunan 3500, Taiwan; kuocc@nhri.org.tw; 9 Department of Family Medicine, Kaohsiung Medical University Hospital, Kaohsiung Medical University, Kaohsiung 807, Taiwan; 10 Department of Post-Baccalaureate Medicine, Kaohsiung Medical University, Kaohsiung 807, Taiwan; rs87203@gmail.com

**Keywords:** gut microbiota, tryptophan metabolism, chronic kidney disease, end-stage renal disease, hemodialysis

## Abstract

Microbiota tryptophan metabolism and the biosynthesis of indole derivatives play an important role in homeostasis and pathogenesis in the human body and can be affected by the gut microbiota. However, studies on the interplay between gut microbiota and tryptophan metabolites in patients undergoing dialysis are lacking. This study aimed to identify the gut microbiota, the indole pathway in tryptophan metabolism, and significant functional differences in ESRD patients with regular hemodialysis. We performed the shotgun metagenome sequencing of stool samples from 85 hemodialysis patients. Using the linear discriminant analysis effect size (LEfSe), we examined the composition of the gut microbiota and metabolic features across varying concentrations of tryptophan and indole metabolites. Higher tryptophan levels promoted tyrosine degradation I and pectin degradation I metabolic modules; lower tryptophan levels were associated with glutamate degradation I, fructose degradation, and valine degradation modules. Higher 3-indoxyl sulfate concentrations were characterized by alanine degradation I, anaerobic fatty acid beta-oxidation, sulfate reduction, and acetyl-CoA to crotonyl-CoA. Contrarily, lower 3-indoxyl sulfate levels were related to propionate production III, arabinoxylan degradation, the Entner–Doudoroff pathway, and glutamate degradation II. The present study provides a better understanding of the interaction between tryptophan, indole metabolites, and the gut microbiota as well as their gut metabolic modules in ESRD patients with regular hemodialysis.

## 1. Introduction

Protein-bound uremic toxins, such as indoxyl sulfate, indole-3-acetic acid (IAA), and indole-3-propionic acid, originate from indole derivatives. These compounds are primarily produced through the indole pathway, which involves the metabolism of tryptophan with the assistance of the gut microbiota. In patients with hemodialysis (HD), the formidable protein-binding capacity of indoxyl sulfate and IAA [1,2] poses a challenge for effective removal through hemodialysis, while also fostering the development of cardiovascular diseases (CVD) in those with end-stage renal disease (ESRD) [3] and contributing to glomerular sclerosis and anemia in CKD groups [4]. Therefore, it becomes imperative to investigate the role of the gut microbiota in the generation of indole derivatives within the HD population.

Tryptophan is an essential amino acid that is ingested only from food such as cheese, red meat, and eggs [5]. Tryptophan has been proven to protect against oxidative stress, memory impairment, and mood disorders in the human body [6]. Tryptophan can be catabolized into serotonin, kynurenine, or indole derivatives [7]. More than 90% of free tryptophan is metabolized to kynurenine and further products via the kynurenine pathway, and the rest can be catabolized to either serotonin or indole derivatives [8]. Indole derivatives are mainly produced by gut indole-producing microbiota such as *Escherichia coli*, *Clostridium* spp., and *Bacteroides* spp. [9]. While the tryptophanase pathway is the most studied route, the comprehensive metabolism of tryptophan still remains unclear, especially for the indole pathway in patients with CKD and ESRD [5,10].

The gut microbiota is a group of bacteria in the gut that plays an important role in the metabolism, immune system, and physiological functions of the host [7]. Research has indicated that individuals with CKD and HD experience dysbiosis-altered gut microbiota [11]. This prompts an exploration into how these changes in the gut microbiota might influence the production of indole derivatives, consequently leading to an escalation in uremic toxins. Tryptophan is catalyzed by tryptophanase and encoded by the tnaA gene, which is abundant in some *Providencia* spp., *Klebsiella* spp., *Shigella* spp., *Proteus* spp., etc. [12,13,14]. The complicated nature of the microbial tryptophan biosynthesis and metabolism pathway (MiTBamp), which involves several enzymes and derivatives in its routes, have also been revealed recently [15]. However, previous studies have mainly focused on tryptophanase and the kynurenine pathway. Consequently, the impact of the gut microbiota on tryptophan metabolism to generate indole derivatives in ESRD patients remains uncertain. Additionally, the field of microbiome research has evolved from utilizing 16S ribosomal RNA gene sequencing to encompass metagenome-wide association studies, centering on the genome shotgun sequencing of the microbiome [16]. This innovative approach not only furnishes insights into the composition of genomes at the species level but also elucidates their functional attributes [17]. Consequently, the primary objective of this current investigation is to elucidate the diversity and signature of the gut microbiota in response to indole metabolite concentrations in ESRD patients undergoing regular hemodialysis, utilizing metagenome-wide genome shotgun sequencing.

## 2. Materials and Methods

### 2.1. Study Population

#### 2.1.1. Study Protocols

The study protocols were approved by the Ethics Committee of Kaohsiung Medical University Hospital (KMUHIRB-E(I)-20160095 and KMUHIRB-E(I)-20180118). All participants provided written informed consent. Hemodialysis (HD) patients were recruited from the dialysis unit of Kaohsiung Medical University Hospital in Taiwan from August 2017 to February 2018. Eligible participants were those who received regular HD three times per week for 3.5–4 h with high-flux dialyzers. Participants with active malignancies or participants who were prescribed antibiotics within three months before enrollment were excluded.

#### 2.1.2. Comorbidities and Laboratory and Clinical Variables

The personal information of patients, including sex, age, medical history, and biochemical data, was collected. DM was identified in patients with HbA1C of 6.5% or higher or taking antidiabetic drugs. Hypertension was identified in patients with a blood pressure of 140/90 mmHg or higher or in patients taking oral antihypertensive medicines. Coronary artery disease was identified in patients with a history of angina and ischemic electrocardiogram change, old myocardial infarction, or in patients who had undergone coronary bypass surgery or angioplasty. Medical history, such as the use of PPI, was also collected.

Blood specimens were collected from an arteriovenous fistula or graft before the participant’s regular HD session with overnight fasting. Biochemical data included albumin. High-sensitivity C-reactive protein (hs-CRP) and albumin samples were collected from routine data within 30 days before the HD session and analyzed in the hospital central lab.

### 2.2. Indole Metabolites Measurement

The tryptophan and related indole metabolites (indoxyl-3-propionic acid, 3-indoxyl sulfate, and indoxyl glucuronide) were measured in this study. The serum samples were mixed with ice-cold acetonitrile containing the internal standard to precipitate proteins. After vortex and centrifuge, the supernatants were transferred to fresh vials. Targeted metabolites were measured using Ultra-performance liquid chromatography (UPLC) coupled with a Xevo^TM^ triple quadrupole mass spectrometer (Waters Corporation, Milford, Massachusetts, United States). In brief, an Acquity UPLC system with a 1.7-μm (2.1 × 100 mm) BEH C_18_ column (1.7 μm, 2.1 × 100 mm) was used to perform liquid chromatography with linear gradient conditions: 0–1.5 min, 1% B; 1.5–2.5 min, 5% B; 2.5–4.5 min, 100% B; 4.5–5.0 min, 100% B; 9.5–12 min, 99% A [solvent system A, water/formic acid (100:0.1, *v*/*v*); B, acetonitrile/formic acid (100:0.1, *v*/*v*)]. The injection volume was 2 μL, and the column temperature was maintained at 35 °C. Mass spectrometric detection was performed on a Xevo triple quadrupole mass spectrometer equipped with an electrospray ionization source operating in positive or negative ionization mode. The source parameters were: capillary voltage, 3.0 kV; cone voltage, 30 V; source temperature, 150 °C; desolvation temperature, 600 °C; cone gas flow, 50 L/h; desolvation gas flow, 800 L/h. The online mass spectrometry analysis used multiple reaction monitoring modes. Waters MarkerLynx XS software version 4.1 (Waters Corporation, Milford, MA, USA) was used for peak detection, alignment, and deconvolution of the UPLC-MS data. Metabolites were identified by matching their accurate masses, retention times, isotope patterns, and MS/MS fragmentation spectra against an in-house library of authenticated standards. Quantification was performed by generating calibration curves using standard solutions of tryptophan, indoxyl-3-propionic acid, 3-indoxyl sulfate, and indoxyl glucuronide.

### 2.3. Microbiome Analysis

#### 2.3.1. Fecal Sample Collection

All stool samples were frozen immediately after collection by each participant, and then delivered in cooler bags to the laboratory (Germark Biotechnology, Taichung, Taiwan) within 24 h.

#### 2.3.2. Shotgun Metagenomics

- Bacterial DNA extraction and metagenomics sequencing

Bacterial DNA was extracted using the QIAamp Fast DNA Stool Mini Kit (Qiagen, Germantown, MD, USA) for stool. The DNA amount and quality were determined with NanoDrop ND-1000 (Thermo Scientific, Wilmington, DE, USA) and agarose gel electrophoresis. The DNA was stored at −80 °C before library construction and sequencing. Extracted DNA (about 500 ng) was fragmented to approximately 350 base pairs using the Covaris S2 system (Covaris, Inc., Woburn, MA, USA) and then subjected to library construction with the Illumina DNA Prep Kit (Illumina, San Diego, CA, USA). Sequencing was performed using an Illumina NovaSeq 6000 sequencer resulting in paired-end reads of 150 bp in length.

- Raw reads quality control

On a per-sample basis, read quality control (QC) was performed using the Kneaddata pipeline (https://github.com/biobakery/kneaddata), which integrates Trimmomatic [18] for trimming Illumina adaptors and low-quality regions, filtering short reads and Bowtie2 [19] for identifying and removing host contaminations from the human hg38 build and PhiX genome. Reads with a low complexity region and repeated sequences were identified and filtered by using the Komplexity software (https://github.com/eclarke/komplexity) with default settings.

### 2.4. Data Analysis

#### 2.4.1. Metagenome Assembly and Annotation

Metagenome assembly was performed by using MEGAHIT [20] to assemble QC-passed reads into contigs. As for taxonomy identification, a contig-based taxonomy analysis was performed, which predicts the taxonomy of contigs using MMSeqs2 [21,22], an easy-to-use taxonomy pipeline, and estimates abundance by contig depth; this is derived by the jgi_summarize_bam_contig_depths script using the bowtie2 alignment mode, which maps QC-passed reads onto contigs. Open reading frame (ORF) prediction and functional annotation [including the Clusters of Orthologous Groups (COGs) family and Enzyme Commission (EC) number assignment] were performed by subjecting contigs into Prokka [23]. ORFs were also analyzed to identify corresponding the KEGG Orthology (KO) number using the MMSeqs2 easy-search pipeline. MinPath [24] was used for pathway reconstruction by analyzing the KO (for KEGG pathway) and EC (for MetaCyc pathway [25]) profiles of each sample. 

Metagenomics binning was performed using MetaBAT [26] to cluster contigs into genome “bins” [i.e., metagenome-assembled genomes (MAGs)]. The quality was assessed and the taxonomy was identified using the CheckM lineage-specific workflow [27]. In addition, the taxonomy of MAGs was performed using GTDB-tk.

#### 2.4.2. Statistical Analysis

We aimed to investigate the relationship between indole pathway metabolite concentration and metagenomic-assembled contig species (MGS) data and genomic metabolic module (GMM) data. Additionally, we examined the influence of demographic and clinical factors on this relationship. The first step was data preparation; we performed several tasks, including importing data and metadata, pruning, aggregating, and grouping data using the R package (version 3.5.1) “phyloseq” [28]. The data were pruned by only selecting certain samples from cases, transforming the MAG dataset sample counts to relative abundance, and removing taxa with a summed abundance of less than 1 × 10^−5^ or those with missing taxonomic information. We then aggregated the data at the species level and created two groups based on the median concentration of a metabolite. Second, we analyzed the study participants’ demographic and clinical characteristics for the high and low metabolite groups. Statistical tests, including *t*-tests, Wilcoxon rank-sum tests, and chi-square tests, were conducted using the “compareGroups” function in the R package (version 3.5.1) to investigate the differences between the groups [29]. 

The contig-based taxonomic profiles were imported and handled by the R package phyloseq [28] and processed for alpha diversity estimation. Beta diversity was analyzed and visualized using principal coordinate analysis (PCoA) via the R package (version 3.5.1) ade4, based on the Bray–Curtis distance of the species-level relative abundance profile [30]. Alpha diversity and PERMANOVA were used to explore differences in microbial community diversity between the high and low metabolite groups. The microbiome data were summarized and visualized with the “ggplot2” package (version 3.5.1) [31]. The “adonis2” function in the R package (version 3.5.1) “vegan” was employed to perform PERMANOVA, and unconstrained ordinations, such as principal coordinate analysis (PCoA), were conducted to visualize the comparison of the microbial community structure in different metabolite levels. Furthermore, we used the Spearman correlation function, adjusted for age and sex as covariates, to explore the association between the relative abundance of MGS and metabolite concentration. Then, we calculated the R-squared values using a linear regression model with MGS selected based on adjusted *p*-values below 0.2 [32]. We also applied Lasso regression to our dataset to identify important microbial taxa associated with the metabolite of interest. We split the dataset into training and testing sets and with a grid search over the tuning parameter to find the optimal lambda value that minimized the mean squared error. Taxa with non-zero coefficients indicated their significant association with the metabolite [33]. Last, we conducted the linear discriminant analysis effect size (LEfSe) [34] and Cladogram analyses to identify taxa that differed significantly in abundance between groups and visualized the phylogenetic relationships between different taxa and their association. In addition, we also used the LEfSe to identify differentially abundant features between high and low metabolite groups in the GMM pathway data [34,35]. Unless otherwise specified, all statistical analyses were performed using R software (version 3.5.1; July, 2018).

## 3. Results

### 3.1. Baseline Characteristics

The baseline characteristics of four metabolites of the indole pathway are summarized in Table 1. The mean age, gender, presence of DM, HTN, CAD, PPI use, Kt/V (D), and hs-CRP levels were similar between the high and low groups for all metabolites. Albumin levels were significantly different between the high and low groups for indoxyl glucuronide (*p* = 0.037), and hemodialysis vintage showed a significant difference for 3-indoxyl sulfate (*p* = 0.003), as shown in Table 1.

### 3.2. Differential Abundance and Diversity of Gut Microbiota in Response to Tryptophan and Indole Metabolite Concentrations

**Figure 1:**
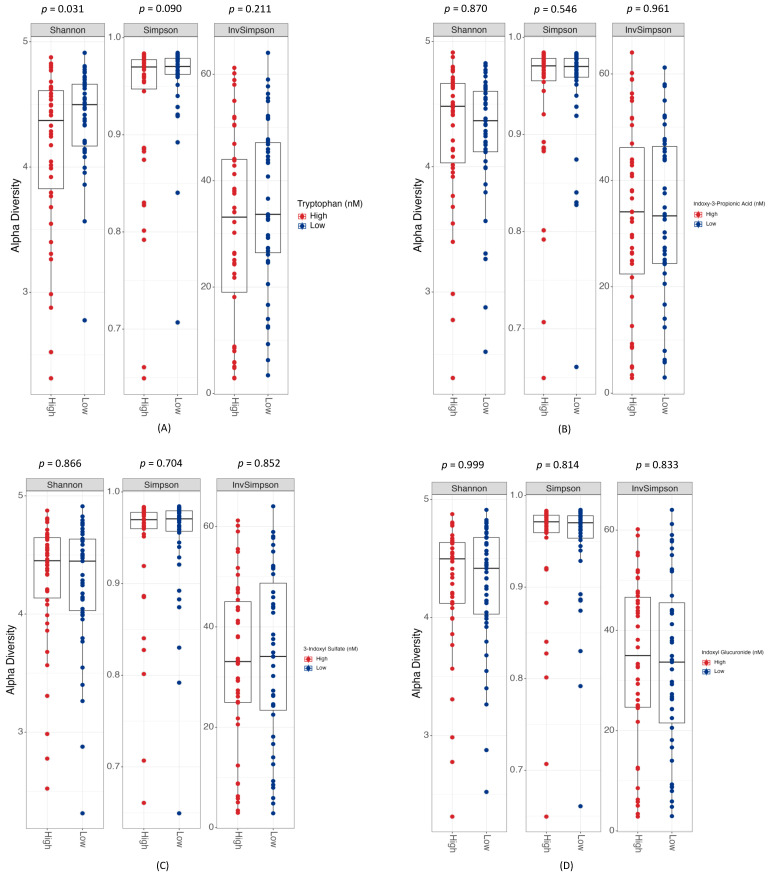
Alpha diversity corresponding to different levels of indole metabolites from the tryptophan pathway: (**A**) tryptophan, (**B**) indoxy-3-propionic acid, (**C**) 3-indoxyl sulfate, and (**D**) indoxyl glucuronide.

We illustrate the differential abundance of genera and species in response to the levels of metabolites along the indole pathway. Each figure underscores the unique microbial distribution influenced by distinct metabolites, specifically the tryptophan metabolite (Supplementary Figure S1), indoxy-3-propionic acid (Supplementary Figure S2), 3-indoxyl sulfate (Supplementary Figure S3), and indoxyl glucuronide (Supplementary Figure S4). The computation of alpha diversity indices, which quantifies the microbial richness and evenness within each patient group, showed that high and low concentrations of tryptophan revealed a significant difference in Shannon diversity (*p* = 0.031), hinting at a distinct community composition between these groups, although the Simpson (*p* = 0.090) and inverse Simpson (*p* = 0.211) indices did not reach statistical significance (Figure 1A). The other indole metabolites, including indoxy-3-propionic acid, 3-indoxyl sulfate, and indoxyl glucuronide, demonstrated no significant difference in Shannon, Simpson, or inverse Simpson indices (Figure 1B–D), suggesting a similar degree of microbial richness and evenness, irrespective of their concentrations.

**Figure 2:**
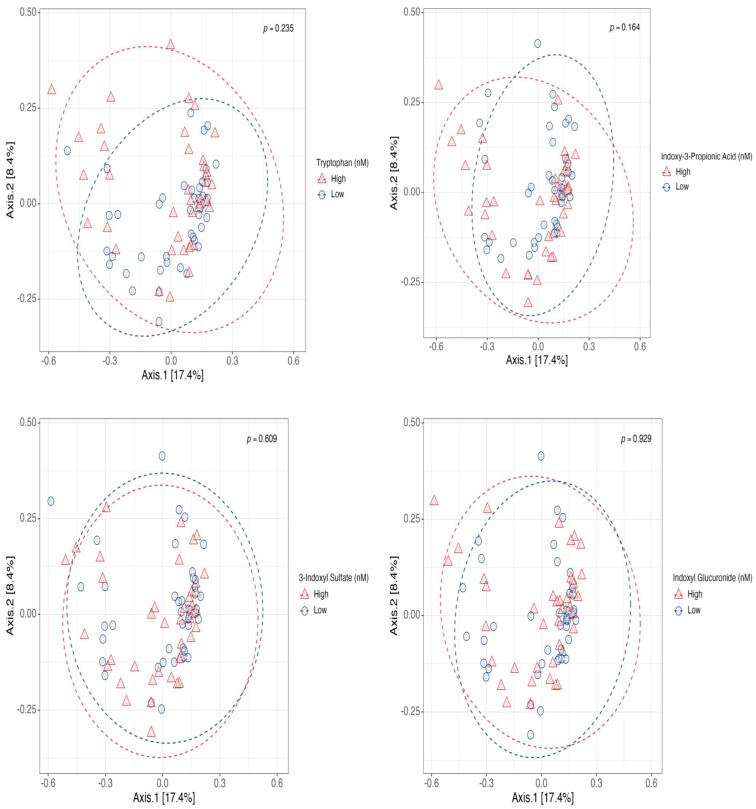
Beta diversity corresponding to different levels of indole metabolites from the tryptophan Pathway: tryptophan, indoxy-3-propionic acid, 3-indoxyl sulfate, and indoxyl glucuronide.

Regarding beta diversity, which captures inter-group dissimilarities, the principal coordinate analysis (PCoA) did not reveal any significant compositional differences associated with varying metabolite concentrations (Figure 2). For tryptophan (*p* = 0.235), indoxy-3-propionic acid (*p* = 0.164), 3-indoxyl sulfate (*p* = 0.609), and indoxyl glucuronide (*p* = 0.929), the community structures appeared similar across different concentration levels, indicating that the concentration of these indole metabolites did not significantly influence the overall microbiota profile. 

### 3.3. Associations between Tryptophan Concentration, Gut Microbiota Composition, and Metabolic Pathways

The relationship between tryptophan and the gut microbiota composition was analyzed by using LEfSe and is shown in Supplementary Figure S5. Among those significantly associated species, a further comparison of the standardized relative abundance between high and low levels of tryptophan is shown in Supplementary Figure S6. Following these observations, as detailed in Table 2, we found that several species, including *Oscillospiraceae bacterium*, *Bacteroides cellulosilyticus*, *Clostridium* sp. CAG:242, *Ruminococcaceae bacterium TF06-43*, *Eisenbergiella tayi*, *[Ruminococcus] torques*, *Bacteroides gallinarum*, and *Oscillibacter* sp. ER4, were associated with the downregulation of tryptophan.

Furthermore, Supplementary Figure S7 demonstrates the distinctive GMMs responding to varying tryptophan concentrations. When tryptophan levels were high, we observed an enrichment in the tyrosine degradation I and pectin degradation I metabolic modules. Conversely, lower tryptophan levels were characterized by the prevalence of glutamate degradation I, fructose degradation, and valine degradation modules.

### 3.4. Influence of Gut Microbiota and Metabolic Pathways on Indoxy-3-Propionic Acid Concentration

The relationship between indoxy-3-propionic acid and the gut microbiota composition was analyzed by using LEfSe and is shown in Supplementary Figure S8. Among those significantly associated species, a further comparison of the standardized relative abundance between high and low levels of indoxy-3-propionic acid is shown in Supplementary Figure S9. Following these observations, we determined, as detailed in Table 2, that specific species, including *Roseburia inulinivorans*, *Roseburia intestinalis*, *Lachnospiraceae bacterium MD329*, *Roseburia* sp., *Sutterella* sp., and *Eubacterium ramulus* were associated with the upregulation of indoxy-3-propionic acid. Conversely, *Ruminococcus gnavus*, *Shigella sonnei*, *Blautia producta*, and *Klebsiella variicola* were linked with its downregulation. These associations, however, were not uniformly significant based on adjusted *p*-values.

Additionally, Supplementary Figure S10 shows the distinct GMMs responding to varying indoxy-3-propionic acid concentrations. High indoxy-3-propionic acid levels were characterized by an enrichment in several metabolic modules, including glutamate degradation I, maltose degradation, and mannose degradation, among others. In contrast, lower concentrations of indoxy-3-propionic acid were associated with metabolic modules such as phenylalanine degradation, glutamate degradation III, and putrescine degradation, showcasing the functional versatility of the gut microbiota in response to changes in indoxy-3-propionic acid levels.

### 3.5. Interplay between 3-Indoxyl Sulfate Concentration, Gut Microbiota Composition, and Metabolic Pathways

The relationship between 3-indoxyl sulfate and the gut microbiota composition was analyzed by using LEfSe and is shown in Supplementary Figure S11. Among those significantly associated species, a further comparison of the standardized relative abundance between high and low levels of 3-indoxyl sulfate is shown in Supplementary Figure S12. This exploration leads us to the critical findings in Table 2, where we identify that *Bilophila wadsworthia* and *Bilophila* sp. 4_1_30 are associated with the downregulation of 3-indoxyl sulfate. However, these associations were not statistically significant based on adjusted *p*-values. Interestingly, we found no species associated with the upregulation of 3-indoxyl sulfate.

Delving further into the functional aspect, Supplementary Figure S13 uncovers distinct GMMs corresponding to the varying 3-indoxyl sulfate concentrations. When the concentration of 3-indoxyl sulfate is high, we observed an enrichment in a handful of metabolic modules, notably alanine degradation I, anaerobic fatty acid beta-oxidation, sulfate reduction, and acetyl-CoA to crotonyl-CoA. Contrarily, lower 3-indoxyl sulfate levels were associated with different metabolic modules, including propionate production III, arabinoxylan degradation, the Entner–Doudoroff pathway, glutamate degradation II, among others. These results illustrate the adaptive function of the gut microbiota responding to the changes in 3-indoxyl sulfate levels.

### 3.6. Interplay between Indoxyl Glucuronide Concentration, Gut Microbiota Composition, and Metabolic Pathways

The relationship between indoxyl glucuronide gut microbiota composition was analyzed by using LEfSe and is shown in Supplementary Figure S14. Among those significantly associated species, a further comparison of the standardized relative abundance between high and low levels of indoxyl glucuronide is shown in Supplementary Figure S15. *Prevotella amnii* and *Dysgonomonas* sp. 511 were identified as having a significant association with the upregulation of indoxyl glucuronide, as indicated in Table 2. No species were associated with this metabolite’s downregulation.

Supplementary Figure S16 demonstrates the differential GMMs reacting to varying concentrations of indoxyl glucuronide. An increase in indoxyl glucuronide concentration was associated with the enhancement of specific metabolic modules, including alanine degradation I, anaerobic fatty acid beta-oxidation, arginine degradation II, propionate production I, succinate conversion to propionate, urea degradation, and arginine degradation V. In contrast, reduced indoxyl glucuronide levels had a distinctive metabolic profile characterized by the prevalence of propionate production III, maltose degradation, hydrogen metabolism, galactose degradation, acetyl-CoA to acetate, xylose degradation, arginine degradation IV, fucose degradation, pyruvate formate-lyase, threonine degradation I, and ethanol production I metabolic modules. This presents a comprehensive picture of how microbial composition and metabolic activity changes align with indoxyl glucuronide concentration variations.

## 4. Discussion

In this present study, we used shotgun metagenomic sequencing to profile the gut microbiota and the functional pathways in ESRD patients with regular hemodialysis. We demonstrated the changes in the gut metabolic modules and composition of gut microbiota responding to varying tryptophan and indole derivative levels. High levels of tryptophan showed a significant decrease in Shannon diversity. To our knowledge, this is the first study to show the interplay between tryptophan metabolism and the biosynthesis of indole derivatives, the gut microbiota, and functional modules in ESRD patients with regular hemodialysis.

### 4.1. Microbiota Composition

The bacteria with a highly conserved tnaA gene can metabolize tryptophan to indole derivatives and utilize tryptophan as energy or for the modification of virulence, such as *Proteus vulgaris*, *Bacteroides thetaiotaomicron*, and *Escherichia coli* [5,13]. Some bacteria also produce indoxy-3-propionic acid, including *Clostridium*, *Akkermansia*, and *Peptostreptococcus* spp., while *Oscillibacter* spp. carry the reductase to catabolize indoxy-3-propionic acid and is negatively correlated with its concentration [36,37,38]. On the contrary, tryptophanase-deficient Bacteroides and other non-indole-producing bacteria, such as *Bifidobacterium*, reduce the levels of indole derivatives [13]. 

In the present study, we found that several species, including *Oscillospiraceae bacterium*, *Bacteroides cellulosilyticus*, *Clostridium* sp. CAG:242, *Ruminococcaceae bacterium* TF06-43, *Eisenbergiella tayi*, *Ruminococcus torques*, *Bacteroides gallinarum*, and *Oscillibacter* sp. ER4, were significantly associated with the downregulation of tryptophan. In previous studies, *Clostridium* spp. and *Bacteroides* spp. were shown to be able to catabolize tryptophan to indole derivatives; this process is negatively related to the tryptophan level [9]. Tryptophan metabolites may further convert to a uremic toxin as well [1,4]. *Eisenbergiella tayi* contains galactosidase, glucosidase, and glucosaminidase, but is not involved in indole production according to previous research [39]. In other studies, the abundance of *Ruminococcaceae bacterium*, *Ruminococcus torques*, and *clostridium* spp. in healthy populations compared to CKD patients showed conflicting results [11,40,41]. Taken together, we found a statistically negative correlation between *Eisenbergiella tayi* and tryptophan level, which may indicate the potential effect of tryptophan catabolism. Furthermore, bacteria with an inverse correlation to tryptophan levels may convert tryptophan into a uremic toxin [42]. Our study offers a possible mechanism for the varying abundance in *Ruminococcaceae bacterium*, *Ruminococcus torques*, and *clostridium* spp. to express their toxicity in ESRD patients. However, the specific mechanism impacting the gut microbiota in ESRD patients still remains unknown and controversial. We also revealed the downregulation of tryptophan in other bacteria for the first time. Konje et al. found a positive association between lower tryptophan levels and CVD incidents in patients with CKD [42]. However, our study did not show a significant difference in CAD and HTN in ESRD patients undergoing regular hemodialysis.

For the concentration of indoxy-3-propionic acid, *Roseburia inulinivorans*, *Roseburia intestinalis*, *Lachnospiraceae bacterium MD329*, *Roseburia* sp., *Sutterella* sp., and *Eubacterium ramulus* are associated with the upregulation of indoxy-3-propionic acid. *Ruminococcus gnavus*, *Shigella sonnei*, *Blautia producta*, and *Klebsiella variicola* are linked with its downregulation. In previous studies, some bacteria that contribute to the upregulation of indoxy-3-propionic acid, such as *Roseburia* spp., some *Eubacterium* families, and *Lachnospiraceae*, were found to be more dominant in non-CKD patients [11,40,41,43,44]. Conversely, *Blautia*, *Klebsiella*, and *Shigella* have a greater abundance in patients with kidney diseases, and a similar association of *Blautia* was also found with adults receiving regular dialysis [44]. Under the anti-oxidative effects of indoxy-3-propionic acid [45], the abundance of those bacteria in non-CKD groups may indicate lower oxidative stress to renal tissue. *Roseburia inulinivorans* and *Roseburia intestinalis* are butyrate producers, which promote gut health [46]. However, *Roseburia* spp. is not specifically known for tryptophan metabolism and indoxy-3-propionic acid production. The *Lanchnospiraceae* family maintains gut health by producing butyrate as well [47]. The oral intake of tryptophan increases the abundance of *Lanchnospiraceae* [8]. The *Eubacterium* genus plays a role in the metabolism of certain dietary polyphenols [48]. It has been found to produce indole-3-acetic acid and join the metabolism of the uremic toxin p-cresol sulfate [49,50]. We demonstrated the positive association between *Eubacterium* and indoxyl sulfate [49]. We found a similar relation between *Eubacterium* and indoxy-3-propionic acid as well. While according to the past research [37,38], indoxy-3-propionic acid is also produced by *Akkermansia*, *Peptostreptococcus*, and some *Clostridium* clusters, we did not observe a positive correlation between indoxy-3-propionic acid and those bacteria. On the other hand, a previous study showed a negative correlation between indoxy-3-propionic acid and *Oscillibacter valericigenes*, *O. ruminantium*, *Butyricicoccus pullicecorum*, *Eubacterium plexicaudatum*, and *Flavonifractor plautii* [38]. We demonstrated the downregulation of indoxy-3-propionic acid to four other species. Among them, *Ruminococcus gnavus* has been associated with gut inflammation and diseases like inflammatory bowel disease (IBD) [51]. Past studies have showed that decreasing tryptophan catabolites resulting from the dysbiosis of gut microbiota were associated with IBD development [50]. Our data provide a reasonable mechanism for *Ruminococcus gnavus* to modify the progression of IBD in the human body. Moreover, we also show the novel interplay of gut microbiota profiles and indoxy-3-propionic acid in ESRD patients undergoing regular hemodialysis.

In the present study, the downregulation of 3-indoxyl sulfate was associated with *Bilophila wadsworthia* and *Bilophila* sp. 4_1_30. *Bilophila wadsworthia* and *Bilophila* sp. 4_1_30 are both part of the *Bilophila* genus of bacteria, which are known to reside in the human gut. *Bilophila* species have been associated with various gastrointestinal conditions, including inflammatory bowel disease and appendicitis, which have been linked to alterations in gut microbial balance or dysbiosis [52]. Moreover, *Bilophila* also has a greater abundance in patients with kidney diseases [44]. As mentioned before, 3-indoxyl sulfate is a uremic toxin that leads to progressive kidney fibrosis, tubular damage, endothelial impairment, and anemia [4]. However, we showed that the downregulation effects of *Bilophila* on 3-indoxyl sulfate though the *Bilophila* were more abundant in patients with kidney diseases. Therefore, other mechanisms may result in the upregulation of 3-indoxyl sulfate in the gut microbiota.

Concerning the indoxyl glucuronide concentration, *Prevotella amnii* and *Dysgonomonas* sp. 511 have a significant association with its upregulation. In previous studies, *Prevotella* was thought to maintain gut glucose homeostasis, but the outcomes conflicted in these studies. Generally, the abundance of *Prevotella* is thought to be positively associated with a non-Western diet [53]. Furthermore, *Prevotella* was found to be enriched in health controls compared to CKD patients [41,44]. However, the upregulation of indoxyl glucuronide from *Prevotella* was shown in our study. Because indoxyl glucuronide is an uremic toxin [54], the decreasing levels of *Prevotella* in CKD or ESRD patients may attenuate the toxicity from this indole derivative. In a rodent model, *Prevotella* spp. was negatively correlated with indoxy-3-propionic acid [38]; however, our study did not reveal a significant correlation between *Prevotella* and indoxy-3-propionic acid. *Dysgonomonas* ferments glucose to produce acid, and it can generate indole as well [55]. However, the definite action of tryptophan metabolism is still not clear. We demonstrated the upregulation of indoxyl glucuronide, which may offer the clear view for the function of *Dysgonomonas*.

### 4.2. Altered Functional Pathways of Gut Microbiota

To our knowledge, this is the first study to show the differences in the metabolic function of the gut microbiota in ESRD patients with regular hemodialysis based on the concentration of tryptophan and its metabolites. Some studies have revealed that functional variations depend on tryptophan in animal models. The comparison of microbiota functions in the human body between CKD patients and healthy controls was also shown in a few studies [11,40,43]. However, the sample size and the number of research studies were limited and lacked consistent outcomes. The present study offers a novel development in this field.

Higher tryptophan levels were associated with tyrosine degradation I and pectin degradation I, while lower tryptophan levels were correlated with glutamate degradation I, fructose degradation, and valine degradation in the present study. Previous studies have showed varying results in different animal models. In a previous study examining a dysbiosis mice model with a high tryptophan diet, an increase in glucose catabolism, glucose uptake, amino acid metabolism, and a decrease in the anaerobic reductive tricarboxylic acid cycle were found [56]. In a fish model, tryptophan feeding resulted in an upregulation of fatty acid biosynthesis, protein digestive absorption, and protein phosphatase I. Conversely, tryptophan was related to the downregulation of phosphofructokinase-2/fructose-2,6-bisphosphatase 3 [57,58]. In macaques, subjects with higher tryptophan levels showed increasing fatty acid biosynthesis, pyruvate fermentation, and glycosyl transferase activity [59]. Our study showed different outcomes regarding protein and carbohydrate metabolism based on varying tryptophan concentrations, though it may not be reasonable to compare the gut microbiota and its functions in different animals. Among the alternations of metabolic function, the upregulation of tyrosine degradation is thought to reduce the essential neurotransmitters in elderly and insect models [60]. Pectin is abundant in fruits and vegetables, and its metabolites promote gut homeostasis [61]. Interestingly, *Monoglobus pectinilyticus*, a member of the *Ruminococcaceae*, is found to have a specific function for pectin degradation [62]. However, our study showed the downregulation between *Ruminococcaceae* and tryptophan concentration. Therefore, other non-specialist bacteria for pectin degradation may be present as well. 

In the present study, the distinct metabolic modules that increased indoxy-3-propionic acid concentrations showed enrichment in glutamate degradation I, maltose degradation, and mannose degradation. In contrast, low indoxy-3-propionic acid is correlated with phenylalanine degradation, glutamate degradation III, and putrescine degradation. Some studies showed the interaction between indoxy-3-propionic acid and lipid–glucose homeostasis, which further promotes insulin sensitivity and reduces lipid toxicity. However, the precise mechanism and its interplay with the gut microbiota remains unknown [63,64]. The upregulation of mannose and maltose degradation from indoxy-3-propionic acid provides a mechanism for the involvement of carbohydrate metabolism. Furthermore, increasing mannose degradation was observed in piglet models treated by the relative abundance of *Ruminococcus*, *Peptococcus*, *Parabacteroides*, etc. [65]. In humans, the promotion of mannose metabolism was also found to increase the abundance of *Prevotella* and *Lachnospiraceae* and decrease *Shigella* [66]. In the present study, *Lachnospiraceae bacterium MD329* was associated with the upregulation of indoxy-3-propionic acid, while *Shigella sonnei* was linked with its downregulation. Our findings may explain the mechanism of mannose degradation from indoxy-3-propionic acid and its interplay with the gut microbiota. Phenylalanine is an essential amino acid and may be converted into tyrosine [67]. Excess phenylalanine is associated with metabolic diseases, heart failure, and a fatty liver [68]. Furthermore, nonalcoholic hepatic steatosis patients with an increased abundance of *Shigella* and decreased *Lanchnospira* showed an upregulation of phenylalanine [68]. We revealed similar outcome based on the interplay between *Lanchnospiraceae* bacterium, *Shigella*, and indoxy-3-propionic acid. Putrescine degradation is associated with nitrogen utilization and GABA metabolism. The metabolic pathway has been well studied in *Escherichia coli* [69]. However, the modulation of putrescine metabolism in indole derivatives and other bacteria remains unclear.

Varying 3-indoxyl sulfate concentrations also modulate the functional aspect of bacteria. Higher 3-indoxy sulfate levels were correlated with the enrichment of alanine degradation I, anaerobic fatty acid beta-oxidation, sulfate reduction, and acetyl-CoA to crotonyl-CoA. On the contrary, lower 3-indoxyl sulfate levels were associated with propionate production III, arabinoxylan degradation, the Entner–Doudoroff pathway, and glutamate degradation II. In previous studies, increasing 3-indoxyl sulfate levels up-regulated the expression of cyclo-oxygenase-2 and inducible nitric oxidase, which leads to oxidant injury to the tissue [70]. The enzyme for alanine degradation produces pyruvate and glutamate, which may further the formation of acetyl-CoA [71]. However, our study revealed upregulation for the route of acetyl-CoA to crotonyl-CoA. This condition may indicate the dysfunction of acyl-CoA dehydrogenase deficiency, which leads to a higher risk of oxidative stress [72]. Furthermore, fatty acid beta-oxidation was also thought to increase lipotoxicity and oxidative stress [73]. Taken together, the altered metabolic function of the gut microbiota based on the increasing 3-indoxyl sulfate levels may explain the mechanism for oxidative damage to the cardiovascular and renal system by this molecule. On the other hand, propionate is the upstream molecule of alanine, which is reasonably associated with a lower 3-indoxyl sulfate level in the present study [71]. Arabinoxylan is a polysaccharide that is rich in dietary fiber and is mainly degraded by *Bacteroidetes* [74]. Furthermore, one arabioxylan-contained molecule was found to inhibit the growth of *Bilophila* in the human body [75]. The interplay of *Bilophila*, 3-indoxyl sulfate, and arabioxylan degradation in the present study may provide another view for their regulation. The Entner–Doudoroff pathway is a process of glucose degradation that produces a source of adenosine triphosphate [76]. Nonetheless, the effect of indole derivatives on that pathway was unclear.

Last, the varying indoxyl glucuronide concentration was involved in a myriad of metabolic pathways. Increased indoxyl glucuronide levels were associated with alanine degradation I, anaerobic fatty acid beta-oxidation, arginine degradation II, propionate production I, succinate conversion to propionate, urea degradation, and arginine degradation V. On the other hand, a decreased indoxyl glucuronide concentration was related to propionate production III, maltose degradation, hydrogen metabolism, galactose degradation, acetyl-CoA to acetate, xylose degradation, arginine degradation IV, fucose degradation, pyruvate formate-lyase, threonine degradation I, and ethanol production I. Generally, the pattern of those variations was similar to 3-indoxyl sulfate. *Prevotella* and *Dysgonomonas*, which are correlated with the upregulation of indoxyl glucuronide, are mainly involved in glucose metabolism in animal models [53,55]. However, our study did not find a significant increase in glucose degradation activity with higher indoxyl glucuronide levels. According to the variant and complicated metabolic pathway alternations with different indoxyl glucuronide concentrations, interactions with multiple bacteria may be involved in the metabolic modules.

Our study has some limitations. First, we did not compare the tryptophan and indole derivative levels in ESRD patients with regular hemodialysis to health controls or CKD patients without dialysis. The significance of different tryptophan metabolites and their effects on health may not be able to be revealed comprehensively. Second, it is hard to directly compare the alternation in the gut microbiota and the variant metabolic functions. Previous studies have demonstrated inconsistent outcomes regarding the microbiota and its function or expression based on environments or the impact of some metabolites. Our data also provided several conflicting or novel results based on past research. Moreover, ESRD may have a significant influence on the gut microbiota composition and genetic modification. As an alternative, we enrolled ESRD patients with regular hemodialysis because there are some different studies focusing on CKD patients. The interplay of the metabolic pathway and the gut microbiota in ESRD patients offers an additional view to develop a better understanding of patients with impaired renal function. Third, we did not test other indole derivatives, such as indole acetic acid or indole-3-aldehyde, which have been studied in the past. However, it is difficult to test all indole metabolites, even if doing so may provide more comprehensive information about microbiota interaction. Studies with a more significant database and the comparison with health controls are needed to determine the importance of tryptophan metabolites and altered gut microbiota in patients with impaired renal function.

## 5. Conclusions

Our study explored the composition and function of microbiome profiles with different concentrations of tryptophan and indole derivatives from ESRD patients. We also highlighted the interplay between tryptophan concentration, indole derivatives, the gut microbiota, and metabolic function in ESRD patients undergoing regular hemodialysis, including a significant difference in Shannon diversity based on the tryptophan concentration. Thus, our research provides valuable insights into these interactions and calls for further investigation into the specific alterations between the gut microbiota, tryptophan metabolites, and metabolic pathways in ESRD patients.

## Figures and Tables

**Table 1:** Demographic and clinical characteristics of study participants in the indole pathway analysis with corresponding metabolite data (*n* = 85).

Parameters/Groups *n* (%) or Mean (SD) or Median [Q1; Q3]		Tryptophan (µM)		*p*	Indoxy-3-Propionic Acid (µM)		*p*	3-Indoxyl Sulfate (µM)		*p*	Indoxyl Glucuronide (µM)		*p*
		High (*n* = 42)	Low (*n* = 43)		High (*n* = 42)	Low (*n* = 43)		High (*n* = 42)	Low (*n* = 43)		High (*n* = 42)	Low (*n* = 43)	
Age		60.0 (10.4)	61.0 (11.1)	0.678	61.0 (10.7)	60.1 (10.9)	0.715	61.1 (10.8)	59.9 (10.8)	0.996	60.5 (11.5)	60.5 (10.1)	0.996
Gender	Female	19 (45.2%)	17 (39.5%)	0.755	19 (45.2%)	17 (39.5%)	0.755	14 (33.3%)	22 (51.2%)	0.149	19 (45.2%)	17 (39.5%)	0.755
DM	YES	10 (23.8%)	18 (41.9%)	0.124	14 (33.3%)	14 (32.6%)	1.000	14 (33.3%)	14 (32.6%)	1.000	14 (33.3%)	14 (32.6%)	1.000
HTN	YES	31 (73.8%)	36 (83.7%)	0.394	32 (76.2%)	35 (81.4%)	0.748	33 (78.6%)	34 (79.1%)	1.000	32 (76.2%)	35 (81.4%)	0.748
CAD	YES	7 (16.7%)	11 (25.6%)	0.459	10 (23.8%)	8 (18.6%)	0.748	12 (28.6%)	6 (14.0%)	0.166	13 (31.0%)	5 (11.6%)	0.056
PPI	YES	6 (14.3%)	7 (16.3%)	1.000	8 (19.0%)	5 (11.6%)	0.516	7 (16.7%)	6 (14.0%)	0.963	7 (16.7%)	6 (14.0%)	0.963
Albumin		3.90 (0.31)	3.92 (0.39)	0.878	3.92 (0.28)	3.90 (0.41)	0.834	3.88 (0.30)	3.94 (0.39)	0.482	3.83 (0.30)	3.99 (0.38)	0.037
Kt/V (D)		1.53 (0.22)	1.54 (0.21)	0.961	1.56 (0.20)	1.51 (0.21)	0.291	1.54 (0.19)	1.53 (0.23)	0.973	1.54 (0.17)	1.53 (0.24)	0.899
hs-CRP		4.97 (5.07)	4.04 (6.54)	0.467	4.13 (4.59)	4.83 (6.89)	0.584	4.39 (6.52)	4.59 (5.19)	0.877	4.21 (5.00)	4.77 (6.66)	0.665
Hemodialysis Vintage		77.4 (63.8)	105 (77.9)	0.074	107 (74.7)	76.8 (67.4)	0.056	115 (78.8)	68.7 (57.5)	0.003	105 (72.4)	78.8 (70.6)	0.100
Metabolites (µM)		42.03 [37.72; 47.56]	28.87 [23.07; 31.32]	<0.001	3.29 [2.13; 5.13]	0.95 [0.48; 1.43]	<0.001	143.72 [11.68; 178.2]	52.88 [40.02; 64.58]	<0.001	1.20 [0.95; 2.39]	0.34 [0.24; 0.48]	<0.001

Note: SD: standard deviation; Q1: the first quartile; Q3: the third quartile; DM: diabetes mellitus; HTN: hypertension; CAD: coronary artery disease; PPI: proton pump inhibitor; hs-CRP: high sensitivity C-reactive protein.

**Table 2:** The associations between signature MGS and metabolites of the indole pathway (*n* = 85).

Metabolites/MGS	T-Statistics	*p*-Values	95% CI
Tryptophan (nM)			
Down Regulate			
*Oscillospiraceae bacterium*	−2.17	0.030 *	[−0.89, −0.04]
*Bacteroides cellulosilyticus*	−2.27	0.030 *	[−0.91, −0.06]
*Clostridium* sp. CAG:242	−2.82	0.006 **	[−1.04, −0.17]
*Ruminococcaceae bacterium TF06−43*	−2.04	0.040 *	[−0.86, −0.01]
*Eisenbergiella tayi*	−2.50	0.010 *	[−0.96, −0.11]
*[Ruminococcus] torques*	−2.86	0.005 **	[−1.04, −0.18]
*Bacteroides gallinarum*	−2.28	0.020 *	[−0.92, −0.06]
*Oscillibacter* sp. ER4	−2.26	0.030 *	[−0.91, −0.06]
Indoxy-3-Propionic Acid (nM)			
Up Regulate			
*Roseburia inulinivorans*	2.70	0.008 **	[0.14, 1.01]
*Roseburia intestinalis*	2.98	0.004 **	[0.20, 1.07]
*Lachnospiraceae bacterium MD329*	3.20	0.002 **	[0.23, 1.12]
*Roseburia* sp.	2.09	0.040 *	[0.02, 0.88]
*Sutterella* sp.	2.43	0.020 *	[0.08, 0.95]
*Eubacterium ramulus*	2.26	0.030 *	[0.05, 0.91]
Down Regulate			
*[Ruminococcus] gnavus*	−2.88	0.005 **	[−1.05, −0.18]
*Shigella sonnei*	−2.02	0.050 ^•^	[−0.86, −0.006]
*Blautia producta*	−2.45	0.020 *	[−0.95, −0.09]
*Klebsiella variicola*	−2.19	0.030 *	[−0.89, −0.04]
3-Indoxyl Sulfate (nM)			
Down Regulate			
*Bilophila wadsworthia*	−2.72	0.008 **	[−1.01, −0.15]
*Bilophila* sp. 4_1_30	−2.48	0.020 *	[−0.96, −0.10]
Indoxyl Glucuronide (nM)			
Up Regulate			
*Prevotella amnii*	1.98	0.050 ^•^	[−0.006, 0.85]
*Dysgonomonas* sp. 511	1.79	0.080 ^•^	[−0.05, 0.80]

Note: ^•^ *p* < 0.1, * *p* < 0.05, ** *p* < 0.01; CI: confidence interval.

## Data Availability

The data will be provided upon request to the corresponding author.

## Supplementary Materials

The following supporting information can be downloaded at: https://www.mdpi.com/article/10.3390/biom14060623/s1, Figure S1: Relative Abundance of Various Genera and Species at Different Levels of Tryptophan Metabolite from the Indole Pathway. Figure S2: Relative Abundance of Various Genera and Species at Different Levels of Indoxy-3-Propionic Acid Metabolite from the Indole Pathway. Figure S3: Relative Abundance of Various Genera and Species at Different Levels of 3-Indoxyl Sulfate Metabolite from the Indole Pathway. Figure S4: Relative Abundance of Various Genera and Species at Different Levels of Indoxyl Glucuronide Metabolite from the Indole Pathway. Figure S5: LEfSe (Linear Discriminant Analysis Effect Size) Analysis and Cladogram Construction for the Microbiome Associated with Tryptophan. Figure S6: The Comparison of Standardized Relative Abundance Between High and Low Levels of Tryptophan. Figure S7: LEfSe (Linear Discriminant Analysis Effect Size) Analysis of Gut Metabolic Modules Based on the Level of Tryptophan. Figure S8: LEfSe (Linear Discriminant Analysis Effect Size) Analysis and Cladogram Construction for the Microbiome Associated with Indoxy-3-Propionic Acid. Figure S9: The Comparison of Standardized Relative Abundance Between High and Low Levels of Indoxy-3-Propionic Acid. Figure S10: LEfSe (Linear Discriminant Analysis Effect Size) Analysis of Gut Metabolic Modules Based on the Level of Indoxy-3-Propionic Acid. Figure S11: LEfSe (Linear Discriminant Analysis Effect Size) Analysis and Cladogram Construction for the Microbiome Associated with 3-Indoxyl Sulfate. Figure S12: The Comparison of Standardized Relative Abundance Between High and Low Levels of 3-Indoxyl Sulfate. Figure S13: LEfSe (Linear Discriminant Analysis Effect Size) Analysis of Gut Metabolic Modules Based on the Level of 3-Indoxyl Sulfate. Figure S14: LEfSe (Linear Discriminant Analysis Effect Size) Analysis and Cladogram Construction for the Microbiome Associated with Indoxyl Glucuronide. Figure S15: The Comparison of Standardized Relative Abundance Between High and Low Levels of Indoxyl Glucuronide. Figure S16: LEfSe (Linear Discriminant Analysis Effect Size) Analysis of Gut Metabolic Modules Based on the Level of Indoxyl Glucuronide.
