# Supplementary material for: Identification of Gut Microbiome Signatures Associated with Indole Pathway in Tryptophan Metabolism in Patients Undergoing Hemodialysis
Source: Biomolecules. 2024 May 24;14(6):623. doi: 10.3390/biom14060623 (PMC11201546; doi:10.3390/biom14060623)

Supplemental Materials

Identification of Gut Microbiome Signatures Associated with Indole Pathway in Tryptophan Metabolism in Patients Undergoing Hemodialysis

Jih-Kai Huang [1,†], Ping-Hsun Wu [2,3,4,5,†], Zhao-Feng Chen [6], Po-Yu Liu [7], Cheng-Chin Kuo [8], Yun-Shiuan Chuang [4,5,9], Meng-Zhan Lu [10], Mei-Chuan Kuo [2,3], Yi-Wen Chiu [2,3] and Yi-Ting Lin [2,4,5,9,*]

[1] Department of Emergency Medicine, Kaohsiung Medical University Hospital, Kaohsiung Medical University, Kaohsiung 807, Taiwan; eric86425@gmail.com
[2] Faculty of Medicine, College of Medicine, Kaohsiung Medical University, Kaohsiung 807, Taiwan; 970392@kmuh.org.tw (P.-H.W.); mechku@kmu.edu.tw (M.-C.K.); chiuyiwen@kmu.edu.tw (Y.-W.C.)
[3] Division of Nephrology, Department of Internal Medicine, Kaohsiung Medical University Hospital, Kaohsiung Medical University, Kaohsiung 807, Taiwan
[4] Center for Big Data Research, Kaohsiung Medical University, Kaohsiung 807, Taiwan; kinkipag@gmail.com
[5] Research Center for Precision Environmental Medicine, Kaohsiung Medical University, Kaohsiung 807, Taiwan
[6] Department of Horticulture and Landscape Architecture, National Taiwan University, Taipei 10617, Taiwan; ivan.chen1966@gmail.com
[7] School of Medicine, College of Medicine, National Sun Yat-sen University, Kaohsiung 804, Taiwan
[8] Institute of Cellular and System Medicine, National Health Research Institutes, Zhunan 3500, Taiwan; kuocc@nhri.org.tw
[9] Department of Family Medicine, Kaohsiung Medical University Hospital, Kaohsiung Medical University, Kaohsiung 807, Taiwan
[10] Department of Post-Baccalaureate Medicine, Kaohsiung Medical University, Kaohsiung 807, Taiwan; rs87203@gmail.com
* Correspondence: emilyhei@gmail.com; Tel.: +886-7-3121101-7021
† These authors contributed equally to this work.

Supplementary Figure S1. Relative Abundance of Various Genera and Species at Different Levels of Tryptophan Metabolite from the Indole Pathway.

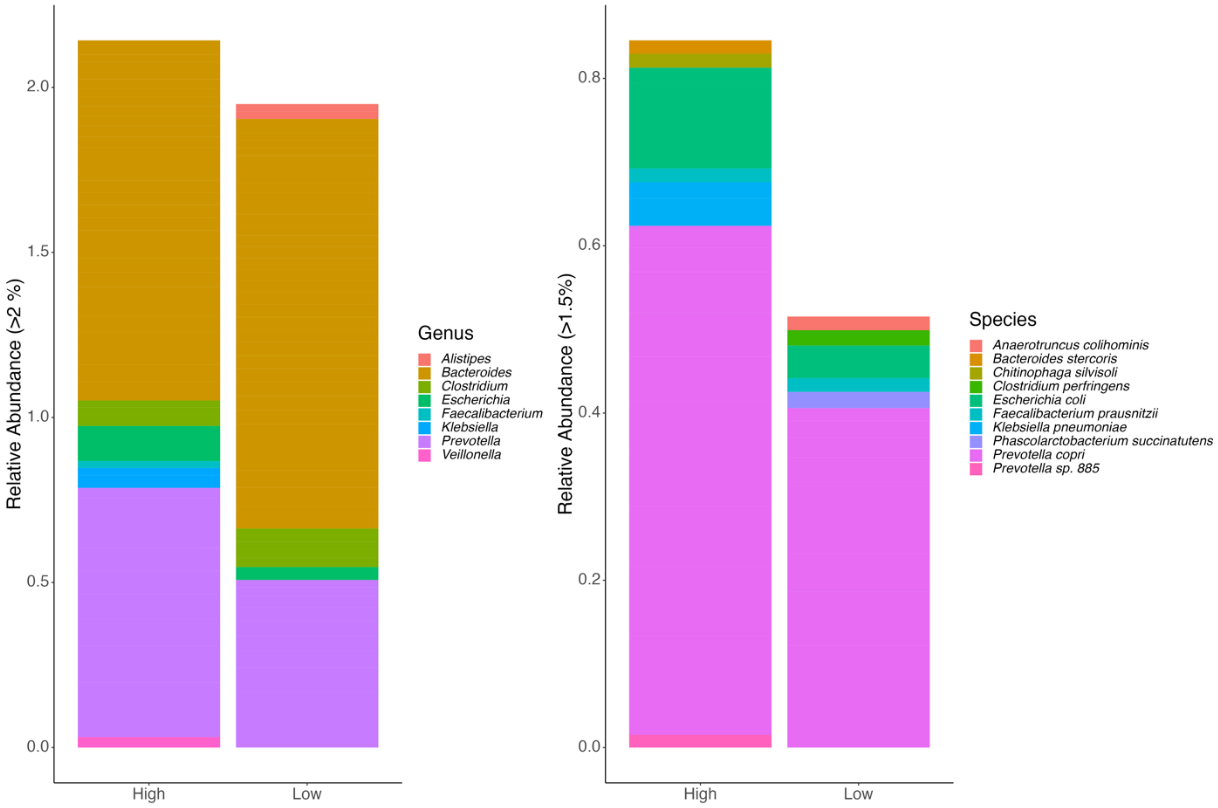

Supplementary Figure S2. Relative Abundance of Various Genera and Species at Different Levels of Indoxy-3-Propionic Acid Metabolite from the Indole Pathway.

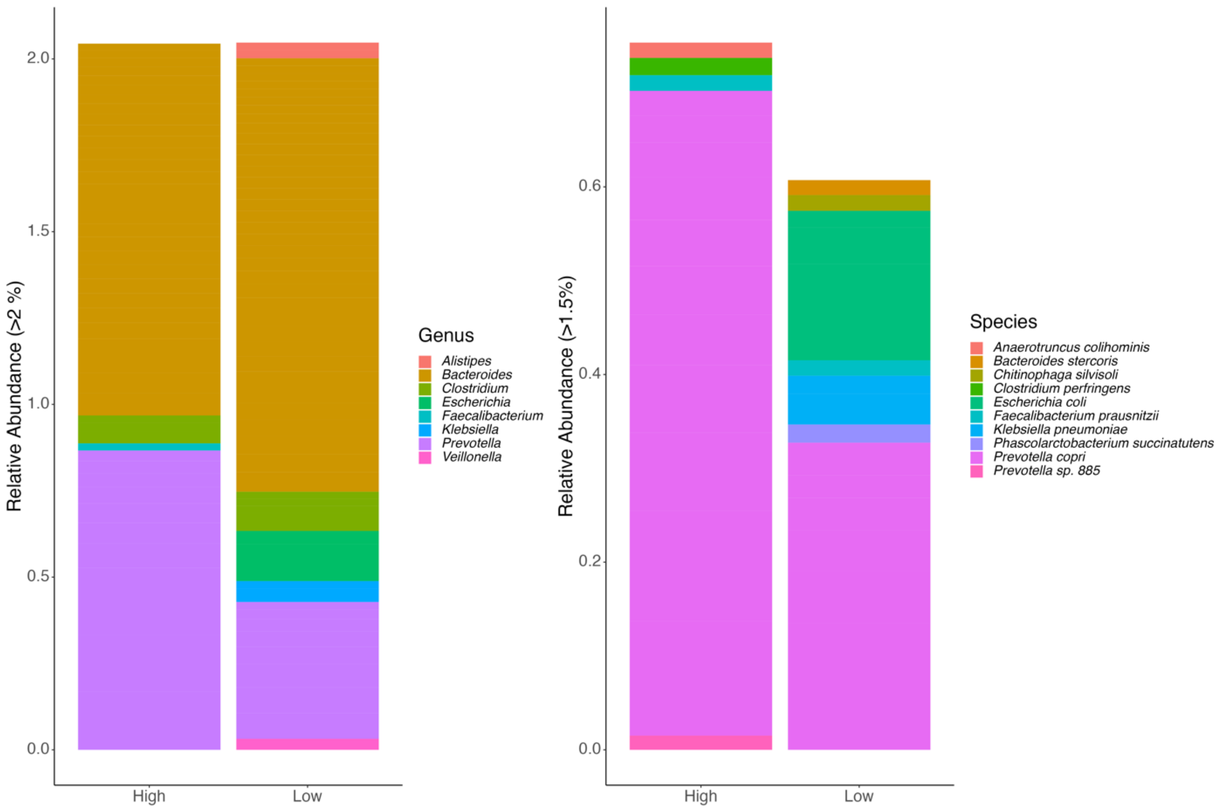

Supplementary Figure S3. Relative Abundance of Various Genera and Species at Different Levels of 3-Indoxyl Sulfate Metabolite from the Indole Pathway.

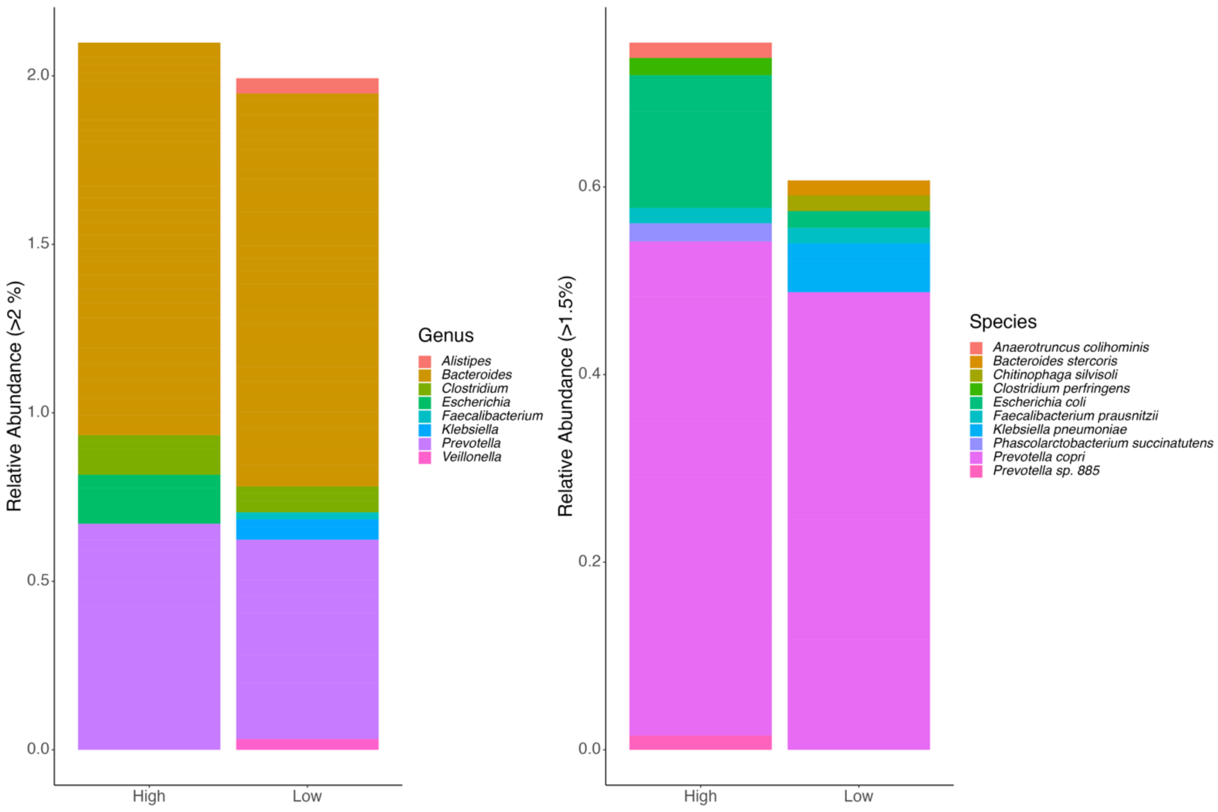

Supplementary Figure S4. Relative Abundance of Various Genera and Species at Different Levels of Indoxyl Glucuronide Metabolite from the Indole Pathway.

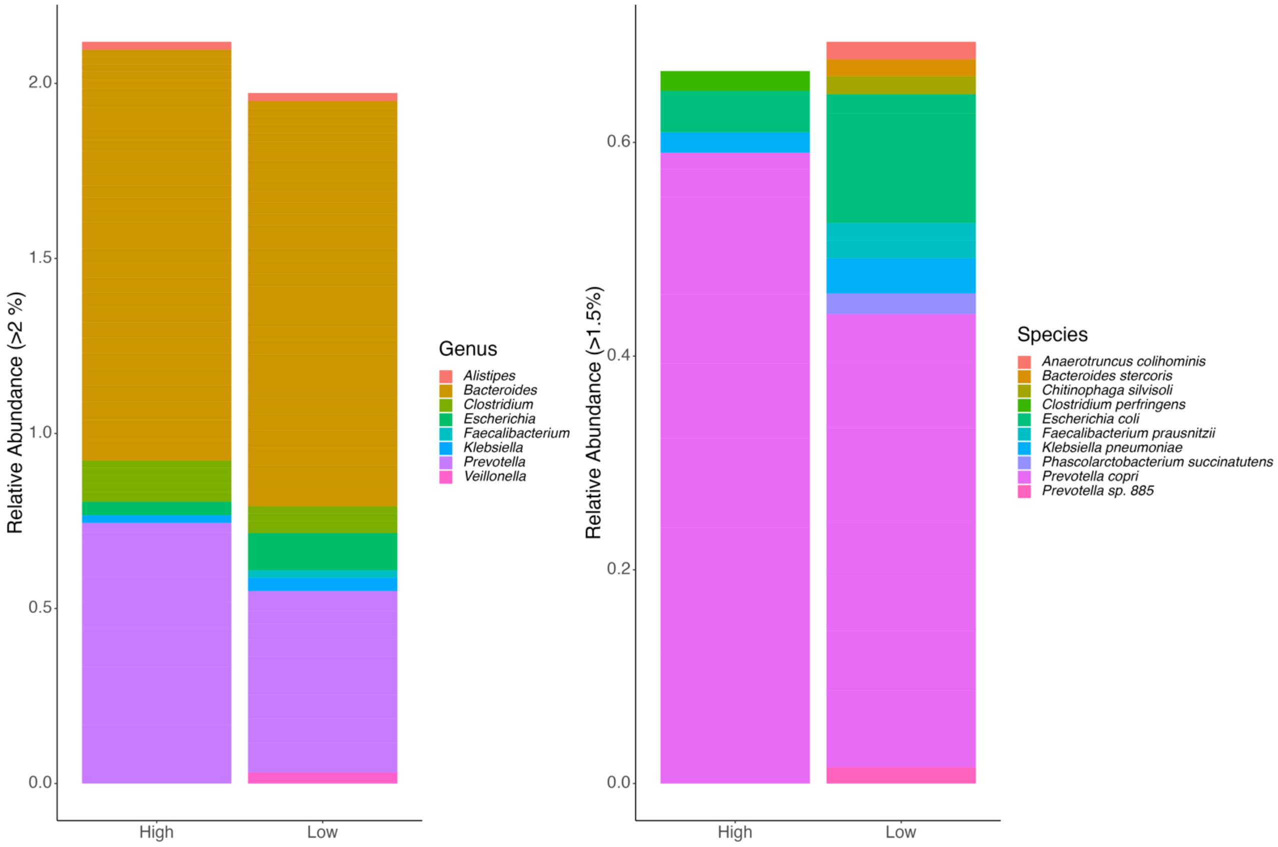

Supplementary Figure S5. LEfSe (Linear Discriminant Analysis Effect Size) Analysis and Cladogram Construction for the Microbiome Associated with Tryptophan.

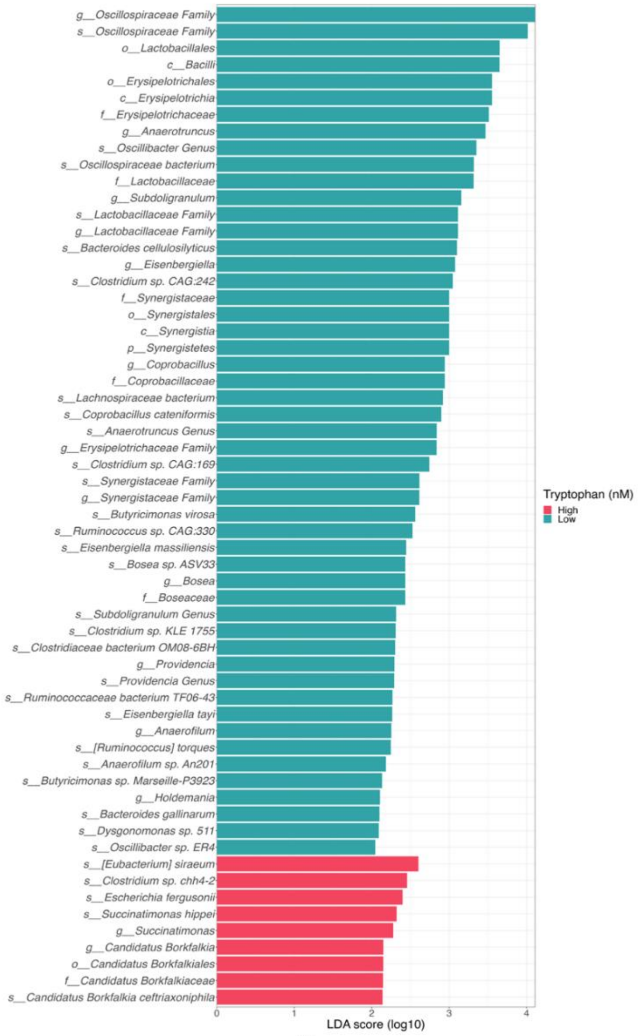

(A)

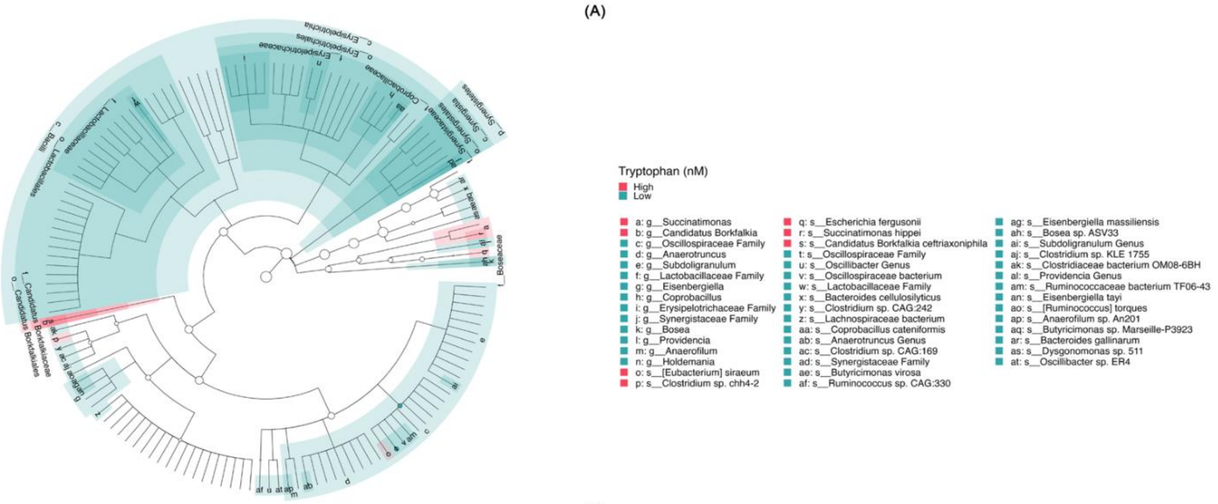

(B)

Supplementary Figure S6. The Comparison of Standardized Relative Abundance Between High and Low Levels of Tryptophan.

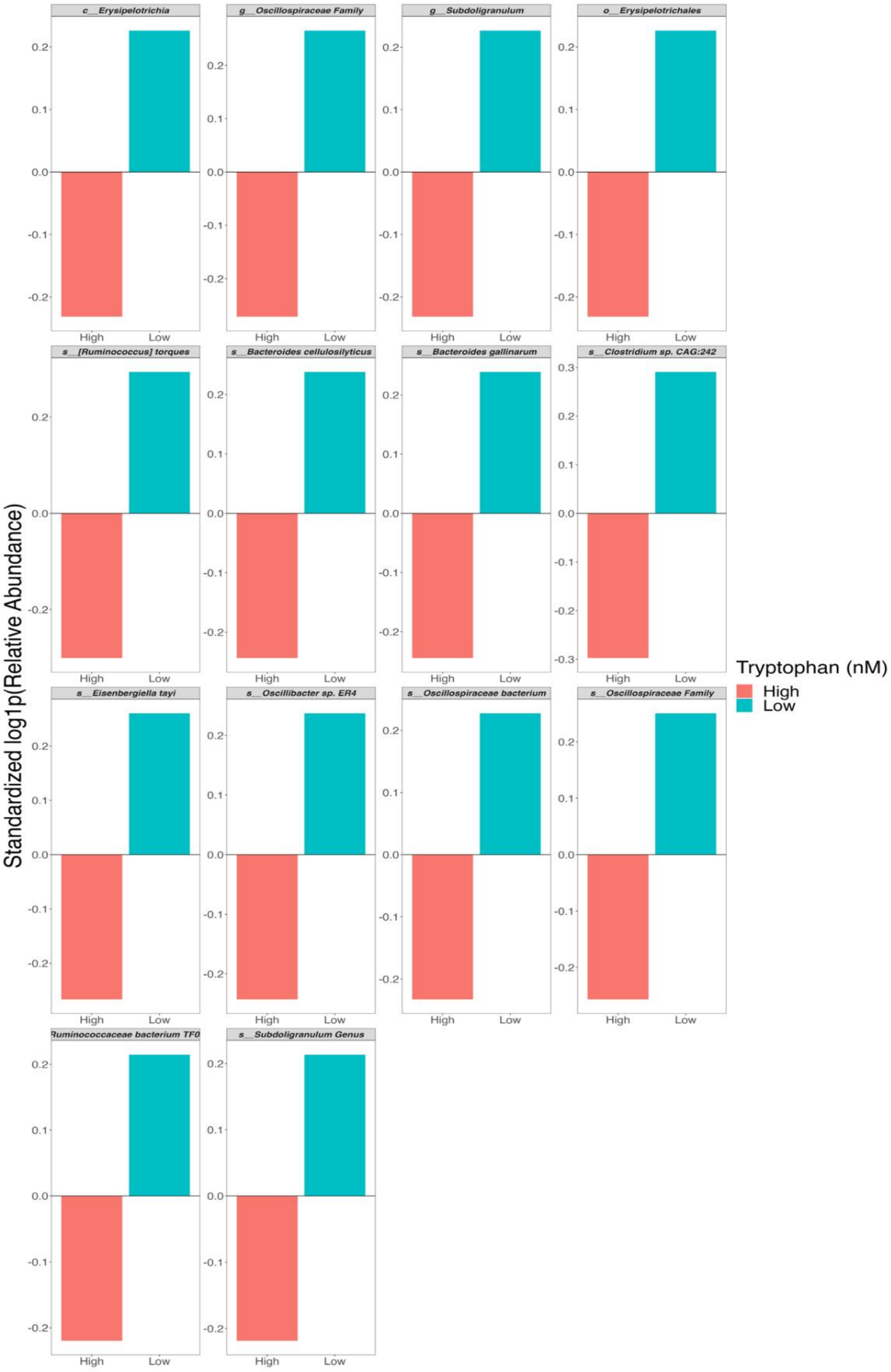

Supplementary Figure S7. LEfSe (Linear Discriminant Analysis Effect Size) Analysis of Gut Metabolic Modules Based on the Level of Tryptophan.

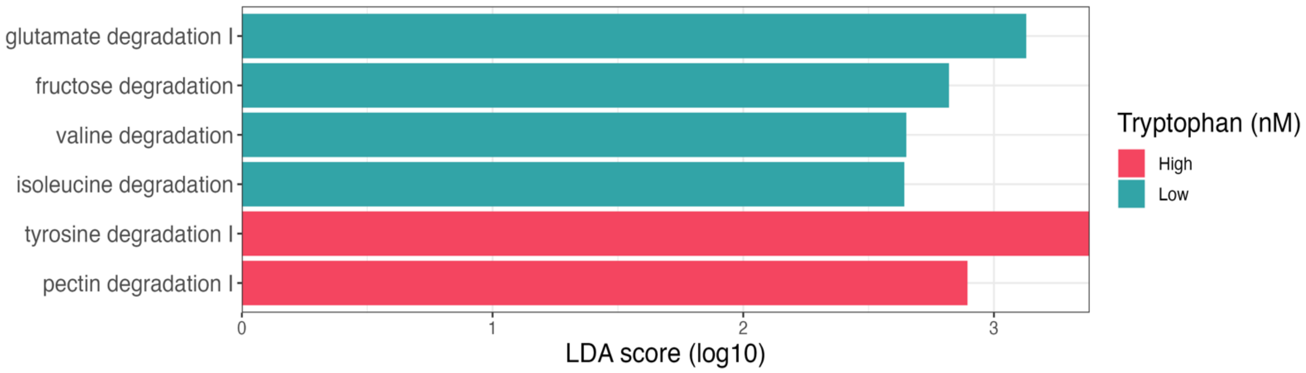

Supplementary Figure S8. LEfSe (Linear Discriminant Analysis Effect Size) Analysis and Cladogram Construction for the Microbiome Associated with Indoxy-3-Propionic Acid.

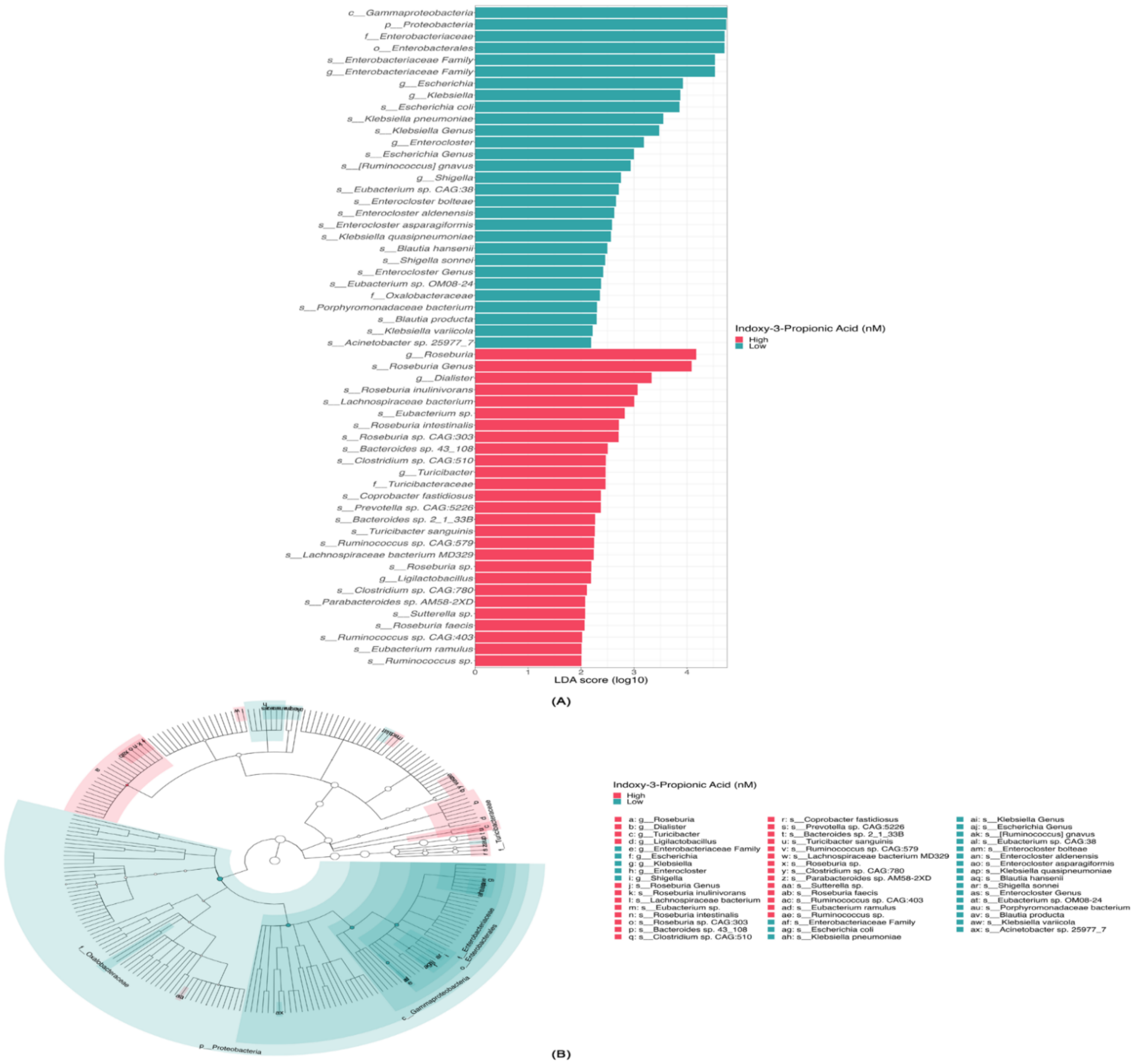

Supplementary Figure S9. The Comparison of Standardized Relative Abundance Between High and Low Levels of Indoxy-3-Propionic Acid.

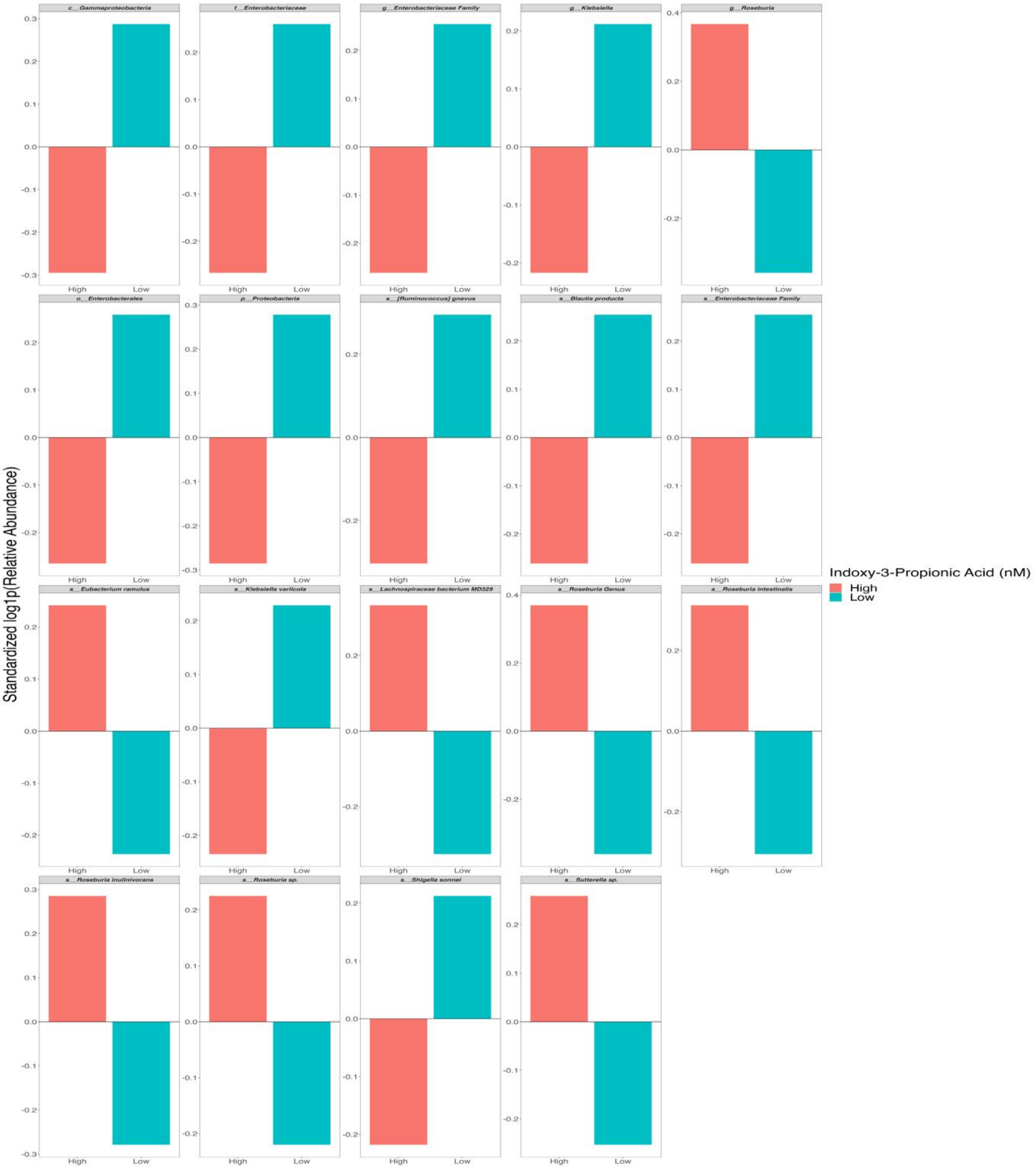

Supplementary Figure S10. LEfSe (Linear Discriminant Analysis Effect Size) Analysis of Gut Metabolic Modules Based on the Level of Indoxy-3-Propionic Acid.

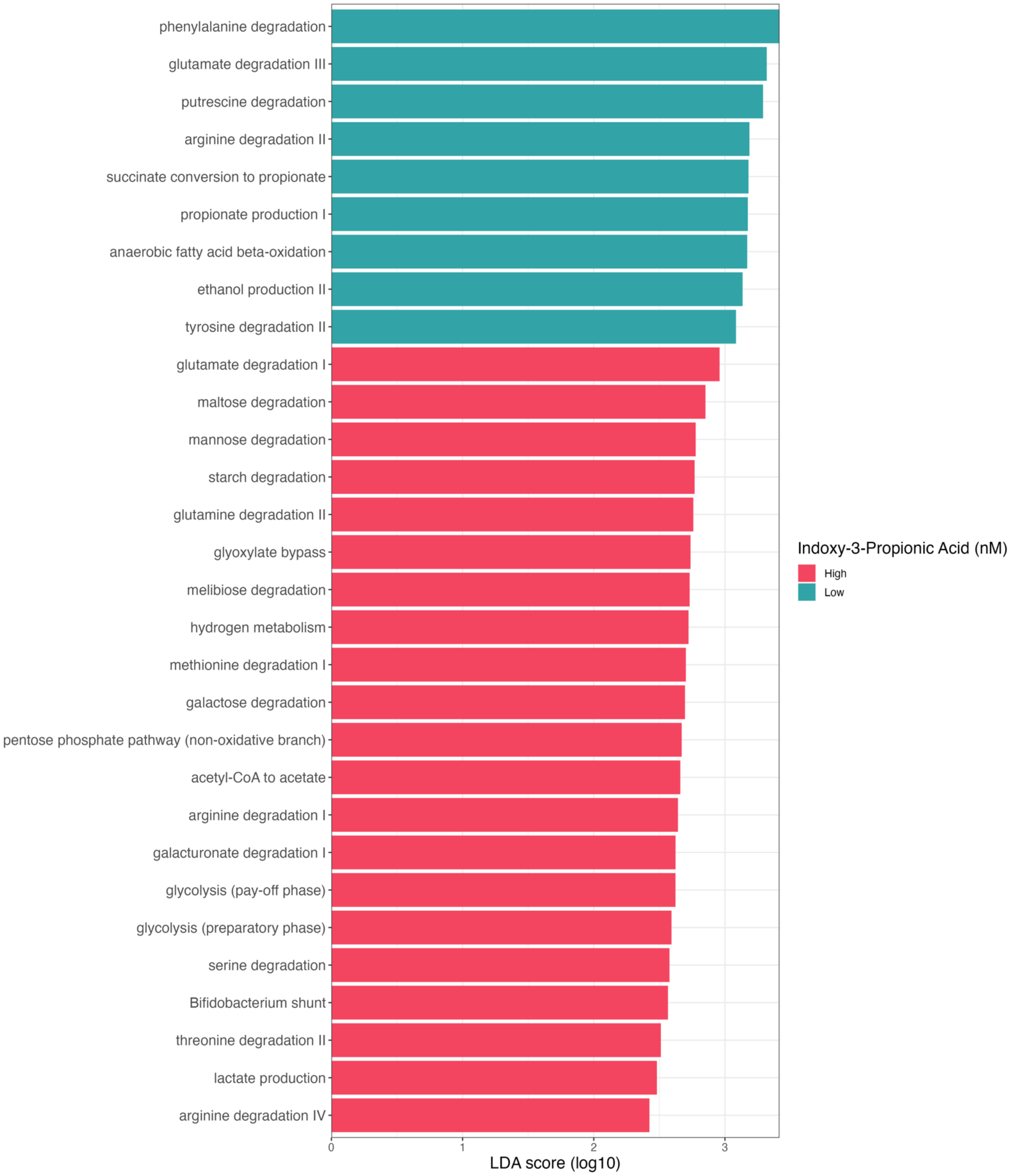

Supplementary Figure S11. LEfSe (Linear Discriminant Analysis Effect Size) Analysis and Cladogram Construction for the Microbiome Associated with 3-Indoxyl Sulfate.

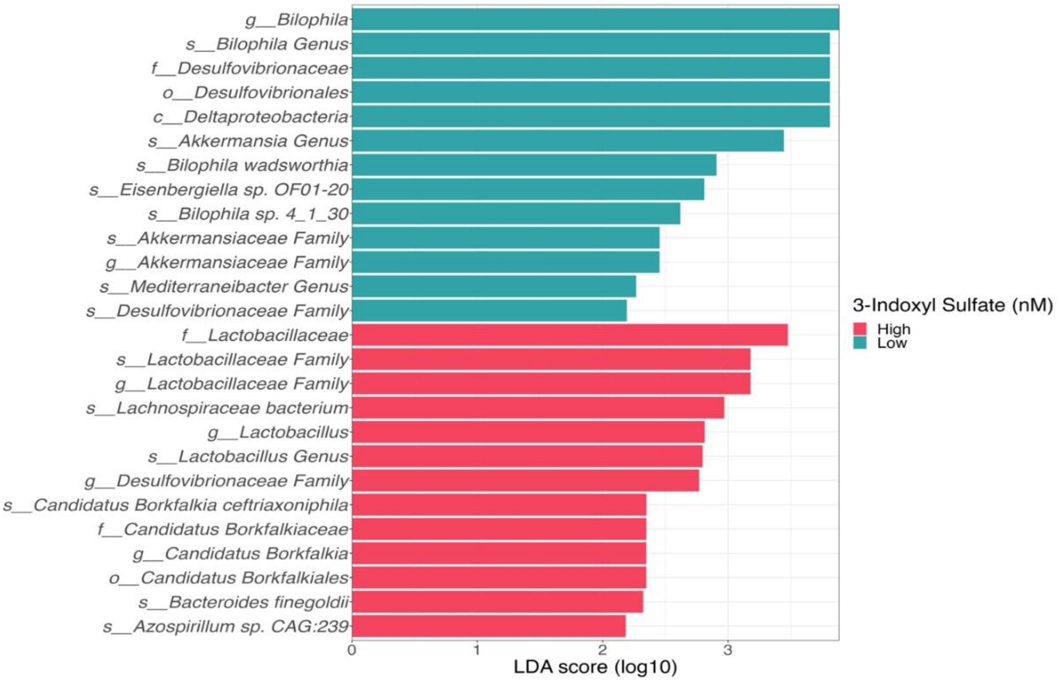

(A)

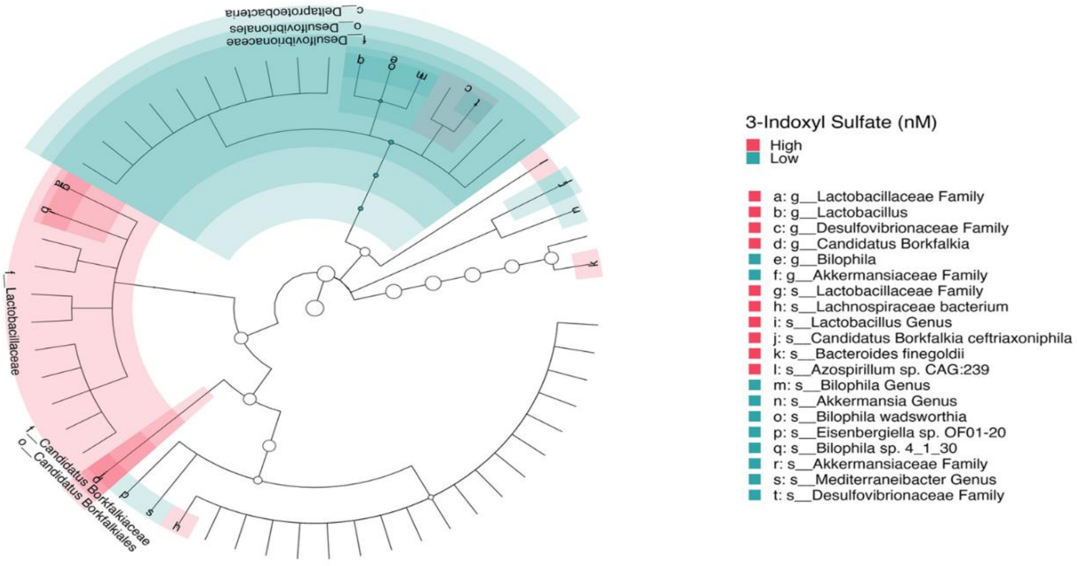

(B)

Supplementary Figure S12. The Comparison of Standardized Relative Abundance Between High and Low Levels of 3-Indoxyl Sulfate.

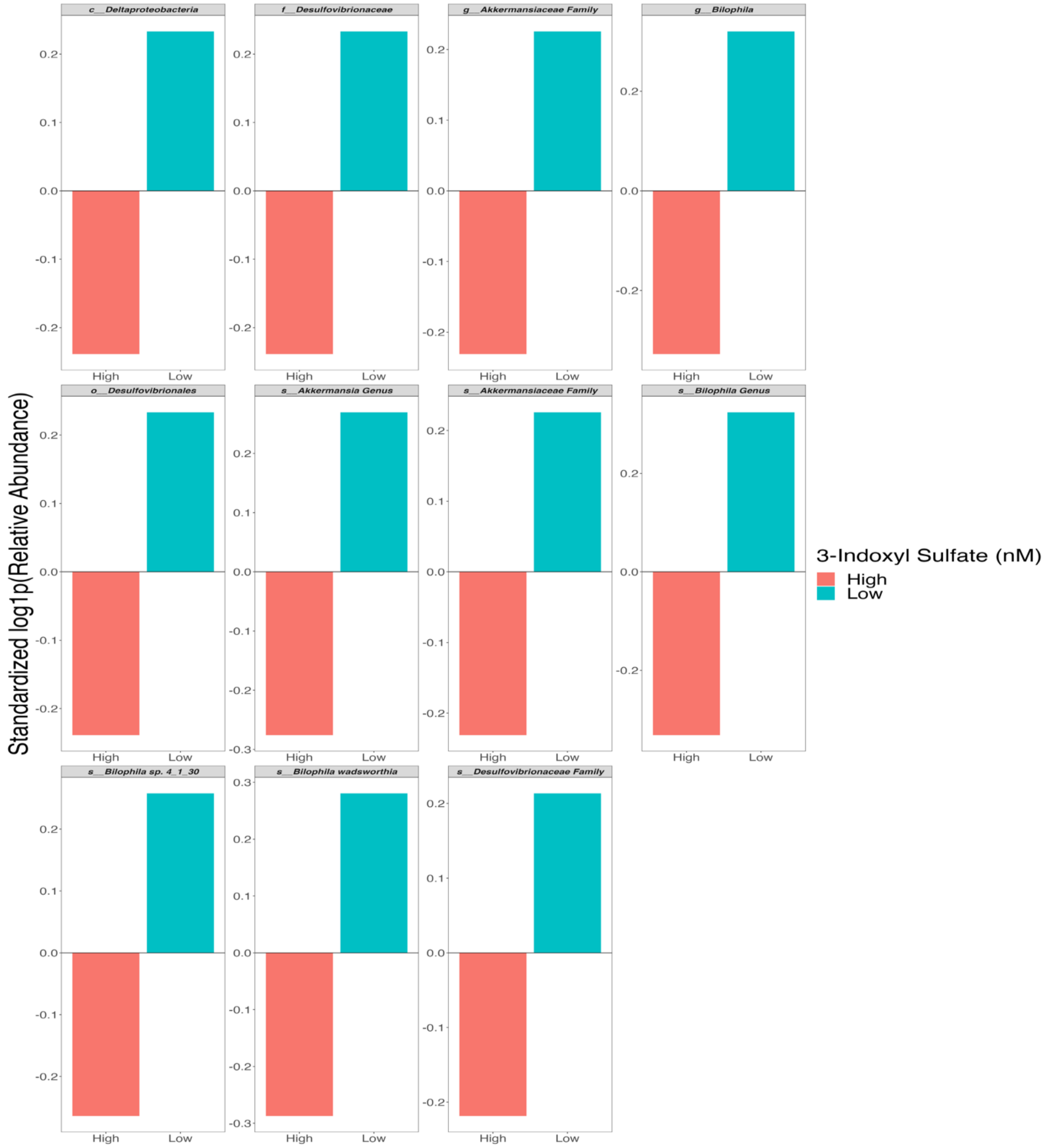

Supplementary Figure S13. LEfSe (Linear Discriminant Analysis Effect Size) Analysis of Gut Metabolic Modules Based on the Level of 3-Indoxyl Sulfate.

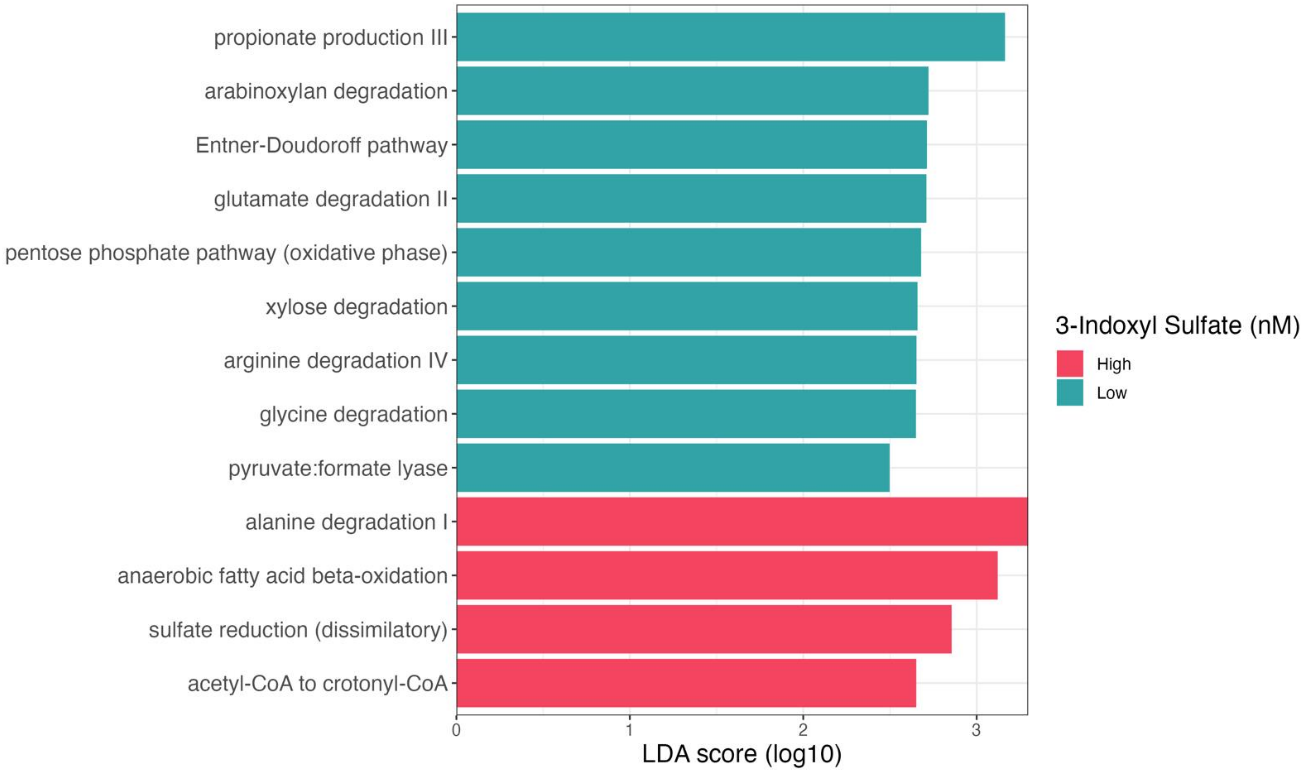

Supplementary Figure S14. LEfSe (Linear Discriminant Analysis Effect Size) Analysis and Cladogram Construction for the Microbiome Associated with Indoxyl Glucuronide.

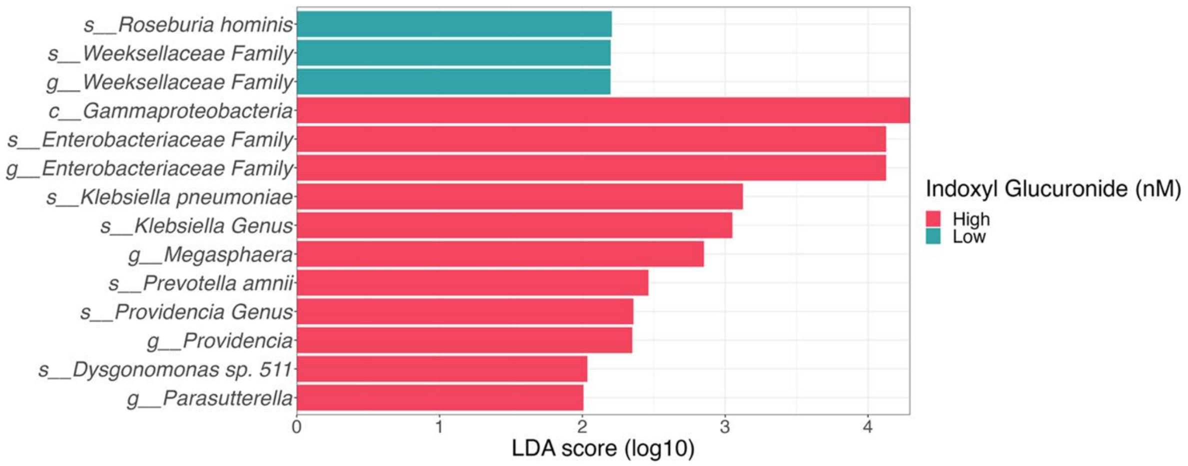

(A)

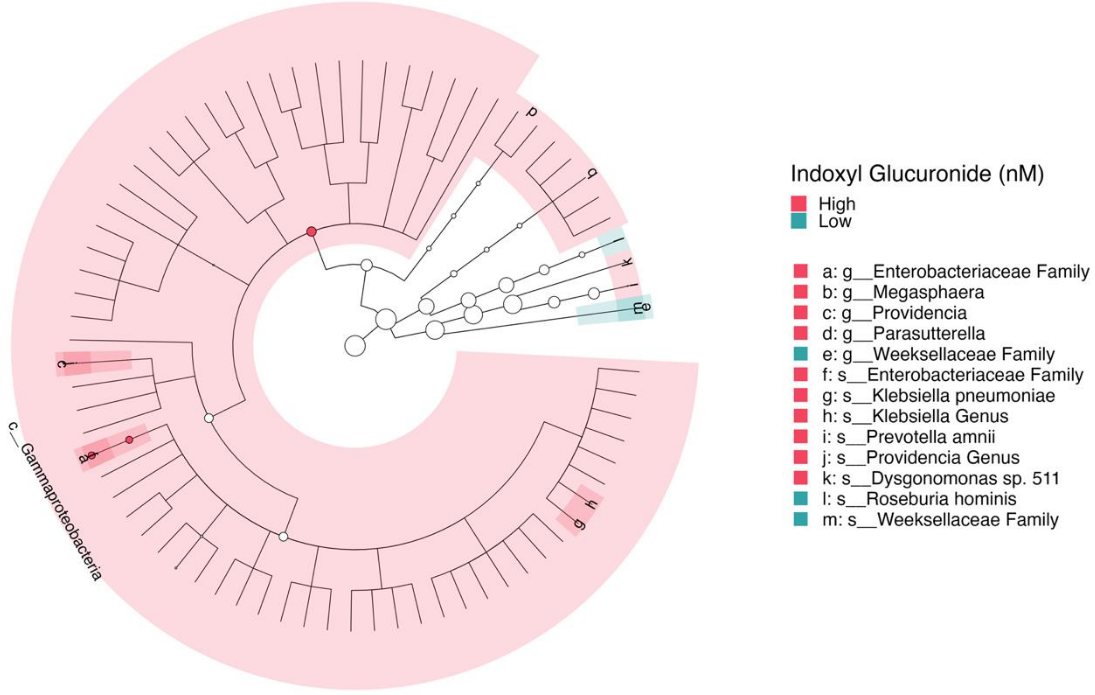

(B)

Supplementary Figure S15. The Comparison of Standardized Relative Abundance Between High and Low Levels of Indoxyl Glucuronide.

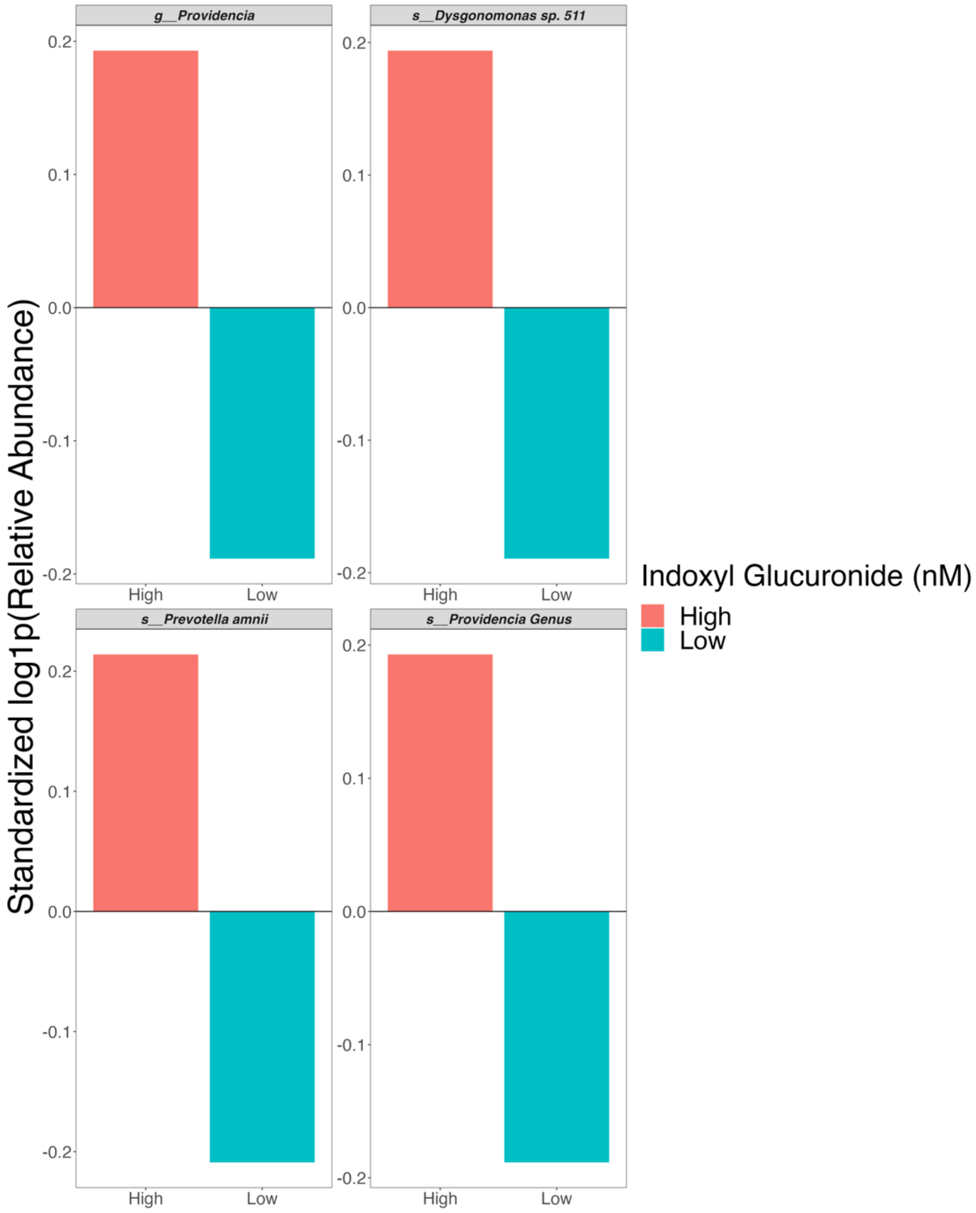

Supplementary Figure S16. LEfSe (Linear Discriminant Analysis Effect Size) Analysis of Gut Metabolic Modules Based on the Level of Indoxyl Glucuronide.

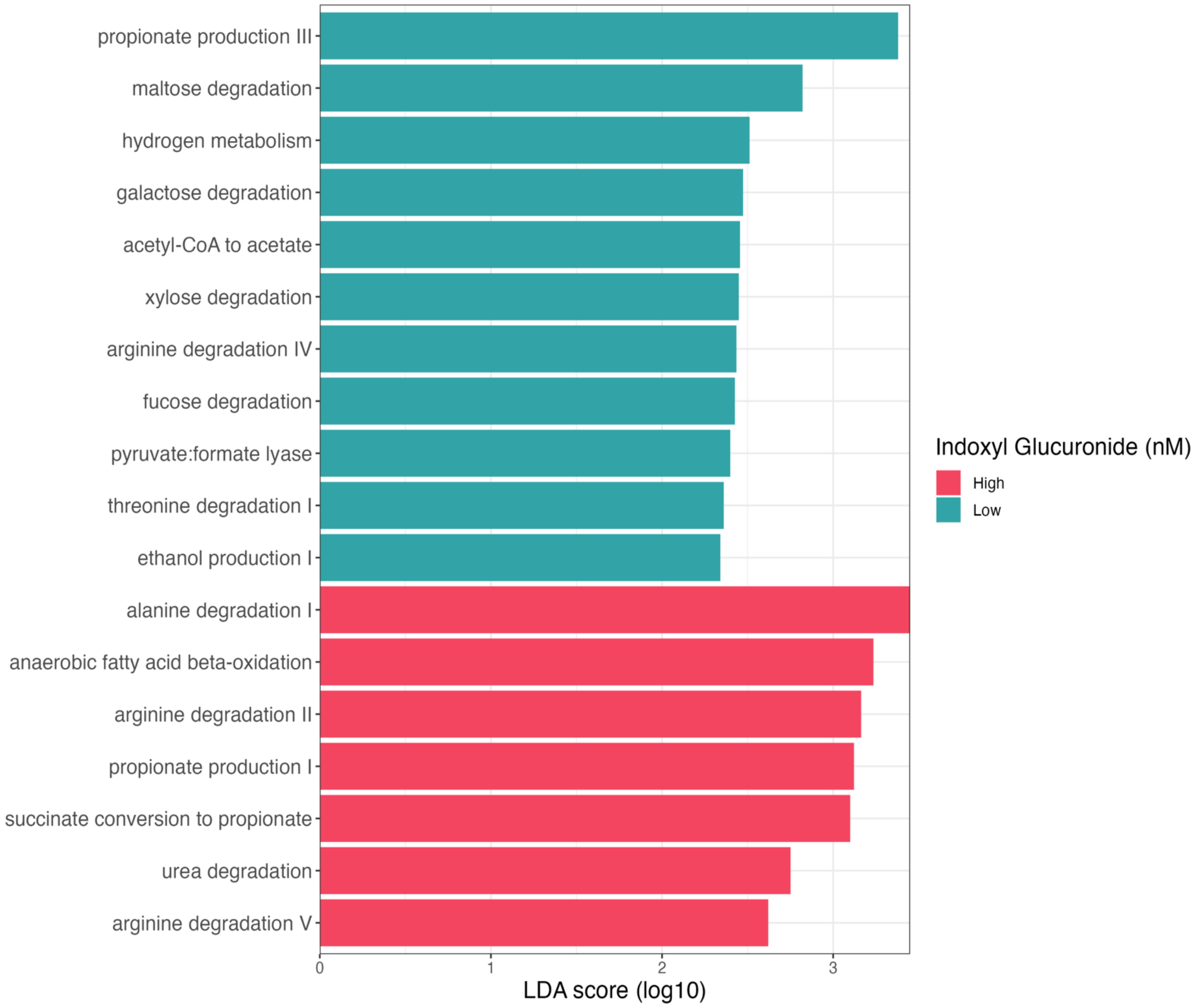

Supplement: Supplementary file 1 [file biomolecules-14-00623-s001.zip › biomolecules-3013304-supplementary.pdf]
